# Brain macrophages acquire distinct transcriptomes in multiple sclerosis lesions and normal appearing white matter

**DOI:** 10.1186/s40478-021-01306-3

**Published:** 2022-01-28

**Authors:** Anneke Miedema, Emma Gerrits, Nieske Brouwer, Qiong Jiang, Laura Kracht, Michel Meijer, Erik Nutma, Regina Peferoen-Baert, Anna T. E. Pijnacker, Evelyn M. Wesseling, Marion H. C. Wijering, Hans-Joachim Gabius, Sandra Amor, Bart J. L. Eggen, Susanne M. Kooistra

**Affiliations:** 1 Department of Biomedical Sciences of Cells & Systems, Section Molecular Neurobiology, University of Groningen and University Medical Center Groningen (UMCG), Groningen, The Netherlands; 2 Faculty of Veterinary Medicine, Institute of Physiological Chemistry, Ludwig-Maximilians-University Munich, Munich, Germany; 3 Department of Pathology, Amsterdam Neuroscience, Amsterdam UMC, VUMC Site, Amsterdam, Netherlands

**Keywords:** Multiple sclerosis, Brain macrophages, Microglia, Single-cell RNAseq, Normal appearing white matter

## Abstract

Multiple sclerosis (MS) is a disease of the central nervous system that is characterized by inflammation and focal areas of demyelination, ultimately resulting in axonal degradation and neuronal loss. Several lines of evidence point towards a role for microglia and other brain macrophages in disease initiation and progression, but exactly how lesion formation is triggered is currently unknown. Here, we characterized early changes in MS brain tissue through transcriptomic analysis of normal appearing white matter (NAWM). We found that NAWM was characterized by enriched expression of genes associated with inflammation and cellular stress derived from brain macrophages. Single cell RNA sequencing confirmed a stress response in brain macrophages in NAWM and identified specific microglia and macrophage subsets at different stages of demyelinating lesions. We identified both phagocytic/activated microglia and CAM clusters that were associated with various MS lesion types. These overall changes in microglia and macrophages associated with lesion development in MS brain tissue may provide therapeutic targets to limit lesion progression and demyelination.

**Supplementary Information:**

The online version contains supplementary material available at 10.1186/s40478-021-01306-3.

## Introduction

Multiple sclerosis (MS) is an auto-immune disease damaging the central nervous system (CNS) and affects 2.8 million people worldwide [1]. MS is characterized by demyelinated lesions in the brain, optic nerves and spinal cord, ultimately resulting in damage to the axons which leads to cognitive problems, blindness, impaired motor function or even paralysis, depending on the location and extent of the lesions [2]. Despite extensive efforts, the factors causing disease and initiating the formation of new lesions are not yet known. Genome wide association studies (GWAS) point towards a role of microglia, the resident macrophages in the parenchyma of the brain, in MS pathology [3]. Besides microglia, other types of macrophages are affected in mouse models for MS, for example perivascular macrophages, meningeal macrophages, choroid plexus macrophages and infiltrating peripheral monocytes, collectively termed CNS-associated macrophages (CAMs) [4].

Additional clues towards the processes involved in lesion initiation are derived from the analysis of the non-lesioned areas from the CNS of affected individuals, such as normal-appearing white matter (NAWM). Compared to healthy controls, NAWM shows several pathological abnormalities [5, 6]. For example, perturbed myelin-axon interactions have been described as an early event in MS NAWM [6]. Additionally, morphologically altered and HLA-DR-expressing macrophages are detected in NAWM [5]. Analysis of gene expression levels indicated that inflammatory processes are ongoing in NAWM and might involve both microglia and astrocytes [7–11]. However, the underlying pathological mechanisms initiating and driving (inflammatory) demyelination in MS remain incompletely understood, and likely involve multiple cell types both within and outside the CNS [12]. To identify early changes in MS brain tissues, we here analyzed the transcriptional profile of NAWM tissues without apparent macrophage (either microglia or CAM) activation (in situ) and applied single-cell RNA sequencing to further determine changes in brain macrophages, including microglia.

## Results

### Transcriptomic changes in normal appearing white matter

**Fig. 1:**
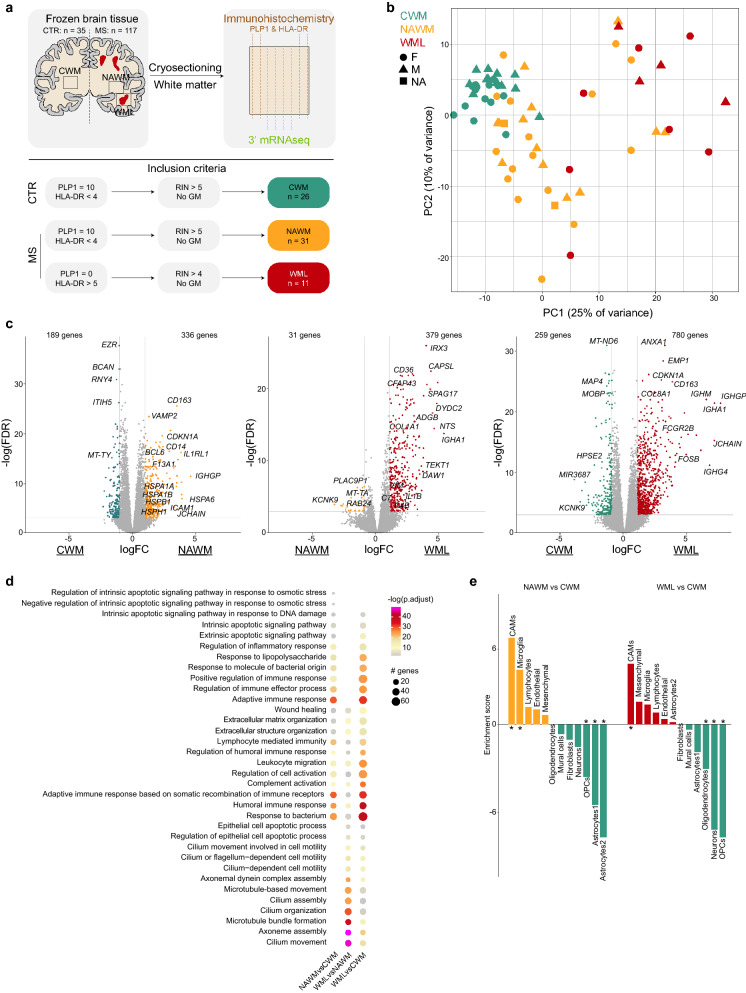
Transcriptomic changes in normal appearing white matter. ** a** Schematic overview of the experiment. **b** Principal component analysis of 68 white matter brain tissue samples. Colors depict donor groups. Shapes depict sex. **c** Volcano plots depicting differential gene expression between NAWM vs CWM samples (left), WML vs NAWM samples (center) and WML vs CWM samples (right). Colored symbols indicate significantly differentially expressed genes (logFC > 1 and adjusted *p *value < 0.05). **d** Dot plot depicting the 10 most significant gene ontology analyses associated with DEGs from the comparisons indicated below. Size depicts number of genes in gene ontology set, color scale depicts significance level. **e** Bar plot depicting expression-weighted gene set enrichment analysis of DEGs between NAWM versus CWM (left) and WML vs CWM (right) based on cell type specific gene signatures derived from human snRNAseq study (Gerrits et al. 2021). *: *p* < 0.05; Abbreviations: CWM = control white matter (CTR donors); NAWM = normal appearing white matter (MS donors); WML = white matter lesion (MS donors)

To identify global disease-associated transcriptomic changes in the brains of MS donors, total tissue transcriptomic profiling was performed on white matter from control donors (CWM), NAWM from MS donors and demyelinated white matter lesions (WMLs) from MS donors (Fig. 1a, Additional file 6: Table S1). To avoid the presence of demyelinated lesions or overt activation of macrophages in NAWM samples, tissue sections immediately adjacent to the tissue sections used for RNAseq were stained for PLP1 and HLA-DR, where for NAWM the PLP1 score had to be 10 to indicate intact myelin and HLA-DR score < 4 representing low-grade macrophage activation status (Fig. 1a, Additional file 1: Fig. S1a, Additional file 6: Table S1). Principal component analysis segregated the WML samples from CWM and NAWM samples in the first principal component (PC1). Furthermore, a clear segregation between NAWM and CWM samples was observed in PC2 and some NAWM samples grouped with WML samples, indicating that the NAWM in MS brains is affected even in the absence of detectable demyelination (Fig. 1b). Differential gene expression analysis confirmed the differences between the groups by identification of hundreds of differentially expressed genes (DEGs; abs(logFC) > 1 and FDR < 0.05) between NAWM vs CWM samples, WML vs NAWM and WML vs CWM (Fig. 1c, Additional file 6: Table S2). Biological processes associated with DEGs enriched in NAWM compared to CWM indicate that apoptosis, stress and inflammatory responses are present in NAWM tissues (Fig. 1d). Additionally, expression of genes reported as immediate-early genes (IEGs), reported to be the first genes being transcribed when cells are stimulated such as during an inflammatory response, was enriched in NAWM compared to CWM (Additional file 1: Fig. S1b, c). DEGs uniquely enriched in WML compared to NAWM were associated with ‘epithelial cell apoptosis’ and ‘cilium assembly and movement’, indicating that these processes occur during or after demyelination/lesion formation. Expression of DEGs associated with ‘extracellular matrix organization’, ‘humoral immune response’, ‘leukocyte migration’ and ‘complement activation’ was enriched in both NAWM and WML samples compared to CWM (Fig. 1d).

To elucidate whether the identified DEGs are caused by changes in cell type composition, we performed expression-weighted cell type enrichment analysis (EWCE) [13]. Expression of DEGs enriched in NAWM when compared to CWM was significantly enriched in CAMs and microglia and depleted in neurons, oligodendrocyte precursor cells (OPC) and astrocytes. Expression of DEGs between WML and CWM was significantly enriched in CNS-associated macrophage (CAM)-genes and depleted in oligodendrocytes, neurons and OPCs (Fig. 1e). These data suggest that these cell types have a different abundance and/or disease-associated transcriptomic changes in gene expression profiles between the conditions.

### Expression of inflammation and stress gene modules is enriched in normal appearing white matter

**Fig. 2:**
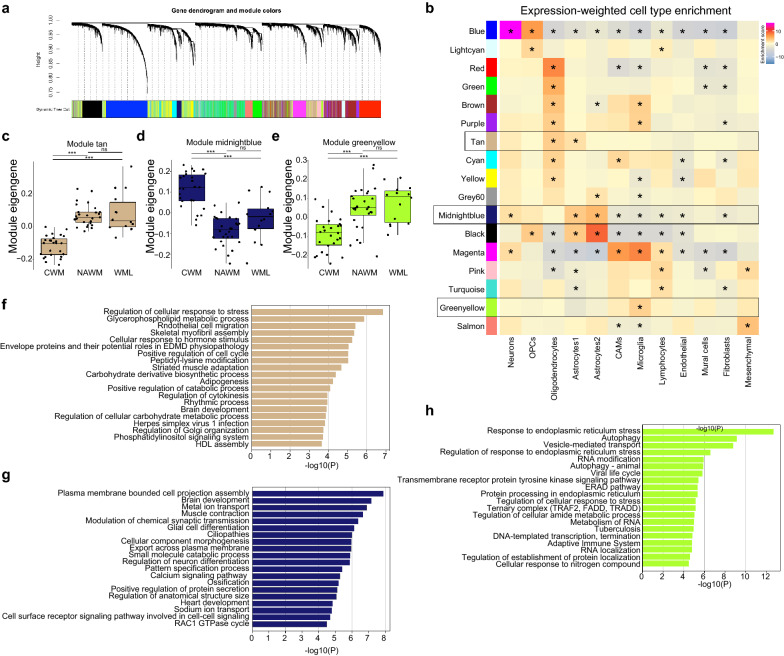
Expression of inflammation and stress gene modules is enriched in normal appearing white matter. **a** Gene dendrogram and module colors of weighted gene correlation network analysis. **b** Heatmap depicting enrichment scores per module of expression-weighted cell type enrichment analysis. Color depicts enrichment score, asterisk indicates significant enrichments (*p* < 0.05). **c–e** Boxplots depicting module eigen genes for the ‘tan’, ‘midnightblue’ and ‘greenyellow’ gene modules. **f–h** Bar plots depicting gene ontology analysis of ‘tan’, ‘midnightblue’ and ‘greenyellow’ gene modules. *: *p* < 0.01; **: *p* < 0.001; ***: *p* < 0.0001. Abbreviations: CWM = control white matter (CTR donors); NAWM = normal appearing white matter (MS donors); WML = white matter lesion (MS donors)

To extract process- or cell type-specific gene modules, a weighted gene co-expression network analysis (WGCNA) was performed (Fig. 2a, Additional file 6: Table S3), and combined with EWCE to identify whether modules contained cell type specific gene expression profiles (Fig. 2b). Expression of the majority of the WGCNA-identified gene modules was enriched in oligodendrocytes, indicating that these cells are amongst the most affected cell types in MS brains, in line with the known pathological features of MS lesions (Fig. 2b). However, not all oligodendrocyte-associated modules were depleted in WML compared to CWM and NAWM (‘red’, ‘green’, ‘brown’, ‘purple’, ‘cyan’, ‘yellow’; Additional file 1: Fig. S1d). This indicates that transcriptomic changes also occur within the oligodendrocyte population, in the absence of loss of oligodendrocyte numbers in WMLs, or that specific subpopulations of oligodendrocytes are lost in WMLs. Additionally, many oligodendrocyte modules were differentially abundant between CWM and NAWM, where no demyelination was present (‘red’, ‘green’, ‘brown’, ‘tan’, ‘cyan’, ‘yellow’; Additional file 1: Fig. S1d). Expression of one such module, ‘tan’, was enriched in NAWM compared to CWM (Fig. 2c) and contained genes enriched in both oligodendrocytes and astrocytes (Fig. 2b). Module ‘tan’ was associated with ‘stress response’, ‘metabolism’ and ‘cell cycle’, suggesting that independent of demyelination oligodendrocytes and/or astrocytes display an early stress response (Fig. 2f). Two gene modules that were enriched in astrocytes were detected (‘black’ and ‘midnightblue’), and both were depleted in NAWM samples compared to CWM (Fig. 2d, Additional file 1: Fig. S1d). Both modules contained genes associated with typical astrocyte functions, indicating that astrocytic support may be depleted in NAWM tissue (Fig. 2g).

Module ‘magenta’, significantly enriched in WML samples, was comprised of genes associated with inflammation, and expression of these genes was enriched in CAMs, microglia and lymphocytes, indicating an increased abundance of these cells in WML or a shift towards a more pro-inflammatory profile (Additional file 1: Fig. S1d, e, Additional file 6: Table S3). Module ‘pink’ also comprised of genes associated with inflammation, and expression of this module was particularly enriched in lymphocytes and in addition corresponded to several GO terms related to inflammation and myeloid cells. Interestingly, module ‘pink’ was also enriched in NAWM vs CWM samples, suggesting that these processes may already be involved in NAWM in MS tissues (Additional file 1: Fig. S1d, e, Additional file 6: Table S3).

Expression of module ‘greenyellow’ was enriched in microglia (Fig. 2b) and was more abundant in NAWM and WML samples compared to CWM. This module was comprised of genes associated with stress response and autophagy, indicative of cellular stress in microglia in NAWM (Fig. 2e, h). These data suggest that the increase in inflammation-, stress- and apoptosis-related gene expression in NAWM and WML total tissue compared to CWM (Fig. 1c, d) is derived from both oligodendrocytes and brain macrophages and occur independent of demyelination.

### Transcriptomic heterogeneity of macrophages in MS brain tissues

**Fig. 3:**
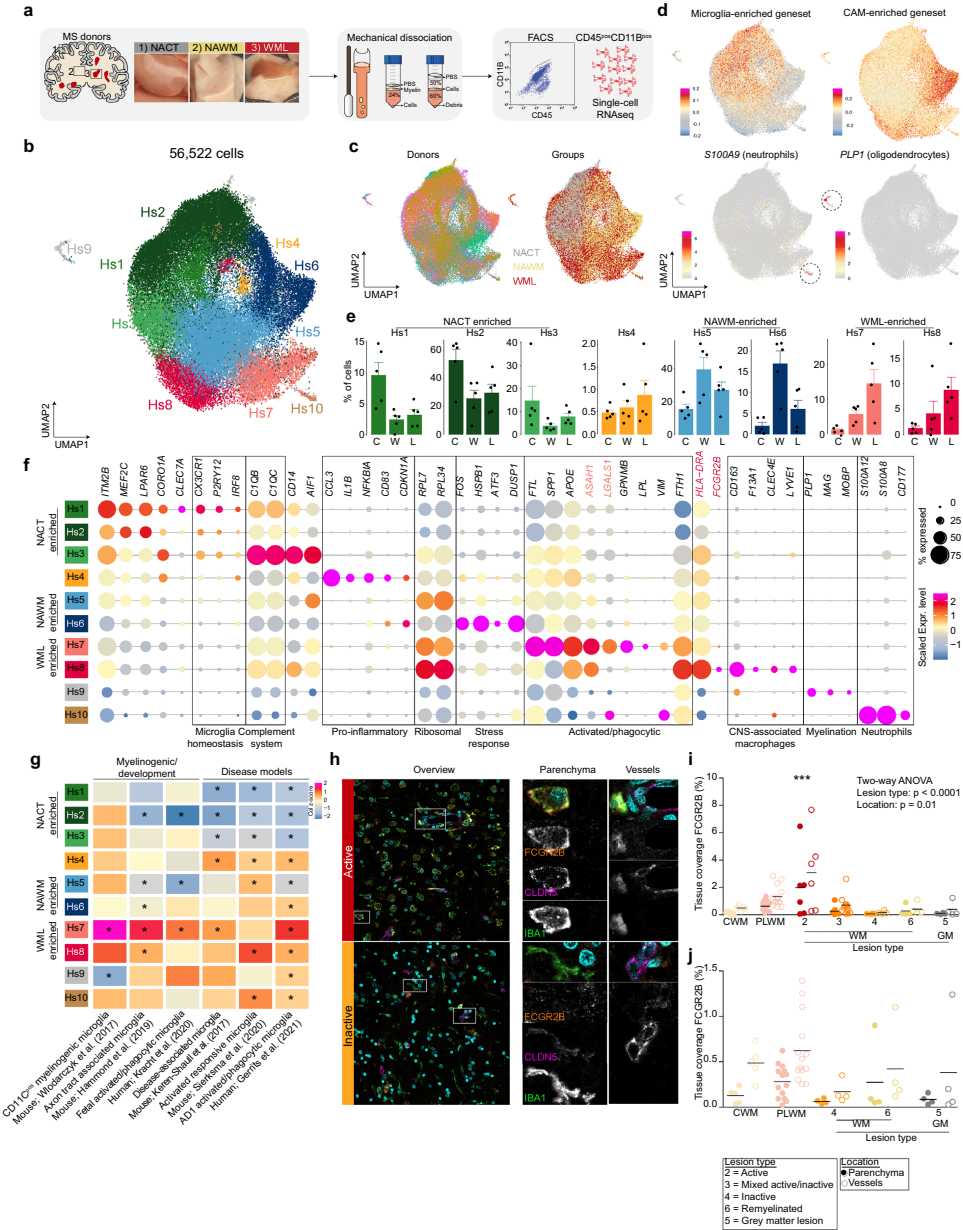
Brain macrophage heterogeneity in multiple sclerosis brain tissues. **a** Workflow of the experiment. **b** UMAP depicting 56,522 cells. Colors depict unsupervised clustering. **c** UMAP where colors depict donor of origin (left) or tissue region of origin (right). **d** UMAPs where color scales depict module scores of microglia- and CAM-enriched gene sets (top) or expression levels of *S100A9* and *PLP1* (bottom). **e** Bar plots depicting cluster distribution among donor groups with standard error. Black circles depict individual samples. C = NACT; W = NAWM; L = WML. **f** Dot plot depicting marker genes of each cluster. Size of the circles depicts the fraction of cells expressing the gene, color scale depicts average expression level. **g** Heatmap depicting enrichment scores of literature-derived gene-sets in the scRNAseq dataset. **h** Immunofluorescent images of stainings for FCGR2B (orange; Hs8); CLDN5 (magenta; blood vessels), Hoechst (cyan; nuclei) and IBA1 (green; macrophages) in an active and inactive lesion. Left panels: overview image. Scale bar = 25 μm. Center panel: Zoom in on parenchymal macrophages. Scale bar = 10 μm. Right panel: Zoom in on vessel-associated macrophages. **i–j** Quantification of immunohistochemistry for FCGR2B in parenchyma and near/on blood vessels. In (**j**) a subset of tissues is presented to enable visualization of differences in samples with low tissue coverage. *: *p* < 0.05. Abbreviations: NACT = normal appearing cortical tissue; NAWM = normal appearing white matter; WML = white matter lesion, PLWM = perilesional white matter

Both stress- and inflammation-related gene expression patterns seemed associated with brain macrophages in NAWM tissue in the bulk RNAseq experiment. Therefore, to investigate whether the detected expression changes were due to altered gene expression patterns within specific macrophage subpopulations in MS brains, scRNAseq was performed on CD45^pos^CD11B^pos^ macrophages isolated from NAWM tissues and tissues with macroscopically visible demyelinated white matter (Fig. 3a). Normal-appearing tissue from the cortex from the same donors was included to obtain sufficient cells with contrasting signatures to identify disease-associated signatures and heterogeneity in NAWM and WML samples whilst avoiding donor variation.

Macrophages were isolated from fresh post-mortem brain tissue within 12 h after autopsy using our mechanical isolation protocol for human microglia [14], performing the entire procedure at 4 °C to minimize possible ex vivo activation of cells (Fig. 3a, Additional file 2: Fig. S2a). In total, 56,522 CD45^pos^CD11B^pos^ cells were analyzed using scRNAseq (Additional file 2: Fig. S2a, b) and these were grouped into 10 subtypes (Hs1-10) using unsupervised clustering (Fig. 3b). None of the clusters was donor-specific, but a segregation based on tissue origin was observed where particularly NACT and NAWM derived cells segregated in the UMAP (Fig. 3c, Additional file 2: Fig. S2c). Significant differences in gene expression between NACT and NAWM-derived cells were observed, where cortical tissue derived cells were associated with ‘phagocytosis’ and ‘synapse pruning’ and white matter macrophages with ‘stress and apoptosis’ (Additional file 2: Fig. S2d, e, Additional file 6: Table S5). The expression of oligodendrocyte and neutrophil genes was enriched in two small clusters (Hs9 and Hs10) and likely represent a small number of non-macrophage cell types derived from the CNS parenchyma and the small amount of blood present in the vessels at the time of autopsy (Fig. 3d, Additional file 6: Table S4).

Recently, we reported the distinct transcriptomic profiles of microglia and CAMs in our single-nucleus RNA sequencing (snRNAseq) dataset of human cortical brain tissues [15]. To distinguish between different types of brain macrophages in the present dataset, gene set module scoring was performed using marker genes specific for microglia or CAMs. Expression of the CAM marker geneset was enriched in clusters Hs6 and Hs8 (Fig. 3d). Expression of typical CAM genes (*CD163*, *F13A1*, *LYVE1* [4]*;*) was enriched in cluster Hs8 and this cluster was most abundant in WML samples (Fig. 3e, f). Cluster Hs6 was particularly enriched in the NAWM samples, whereas cluster Hs8 was enriched in WML samples (Fig. 3e). The identified gene signatures for the human macrophage subtypes we identified was comparable with macrophage/microglia subsets identified in other studies (Fig. 3g). Direct comparison of the transcriptomes of Hs6 and Hs8 cells showed that Hs6 cells are mainly associated with stress response and changes in oxygen levels, whereas Hs8 associated with inflammation and antigen presentation (Additional file 3: Fig. S3a, b). In MS, distinct lesion types can be identified that are characterized by different degrees of demyelination and inflammation [16]. Hs8 is characterized by high expression of FCGR2B, that has been previously described to be differentially expressed between microglia and perivascular macrophages in MS lesions. It has been suggested that expression of Fc receptors on microglia and perivascular macrophages may be highly relevant to the pathogenesis of MS [17]. Immunohistochemistry/fluorescence for the proteins FCGR2B and HLA-DR in five MS lesion types, showed that Hs8 macrophages are scarce in non-active lesions and almost exclusively found in and in close proximity to blood vessels, as confirmed by co-localization with CLDN5 (Fig. 3h–j). Conversely, in active MS lesions, IBA1^pos^FCGR2B^pos^HLA-DR^pos^ cells (Hs8) were much more abundant in both blood vessels and the parenchyma (Fig. 3h, i). We also found that these cells are positive for CLDN5, as has been previously observed for monocytes in the context of MS [18]. These data indicate that macrophages and/or monocytes with a CAM-like signature infiltrate the brain parenchyma during active lesion formation or that parenchymal microglia adopt a CAM-like signature.

Expression of heat-shock-protein (HSP) genes, IEGs and genes associated with stress response was enriched in cluster Hs6. Since these findings are in line with the enrichment of HSP-genes and IEGs in total tissue NAWM compared to CWM (Fig. S1b), this strongly suggests that HSP gene and IEG expression of macrophages in NAWM compared to NACT is not an intrinsic property of WM macrophages, but rather is MS pathology associated.

Expression of microglia marker genes was enriched in the majority of the cells, but depleted in clusters Hs6, Hs7 and Hs8 (Fig. 3d). Expression of typical homeostatic microglia markers including *CX3CR1*, *P2RY12 and IRF8* was enriched in clusters Hs1, Hs2, Hs3 and Hs5, suggesting that these clusters represent homeostatic microglia (Fig. 3f, Additional file 6: Table S4). Expression of genes associated with the complement system was enriched in cluster Hs3 (Fig. 3f). This cluster was most abundant in NACT samples, but also slightly enriched in WML compared to NAWM (Fig. 3e, Additional file 2: Fig. S2c). The expression of pro-inflammatory genes was enriched in cluster Hs4, this cluster was equally abundant in all groups (Fig. 3e, Additional file 2: Fig. S2c). Expression of myelinogenic, developmental and disease-associated microglia genes (*FTL*, *SPP1*, *ASAH1* and *GPNMB*), that have activated/phagocytic microglia phenotypes in mice, was enriched in cluster Hs7 (Fig. 3f, g, Additional file 6: Table S4). These data indicate the cells in cluster Hs7 are microglia, associated with demyelination or other myelin-associated processes.

Overall, the identified gene signatures for the human macrophage subtypes we identified in this study were comparable with macrophage/microglia subsets identified in our previous snRNAseq study (Gerrits et al., 2021), indicating robust microglia subtyping in these studies (Additional file 3: Fig. S3c).

### Activated/phagocytic microglia arise during demyelination

**Fig. 4:**
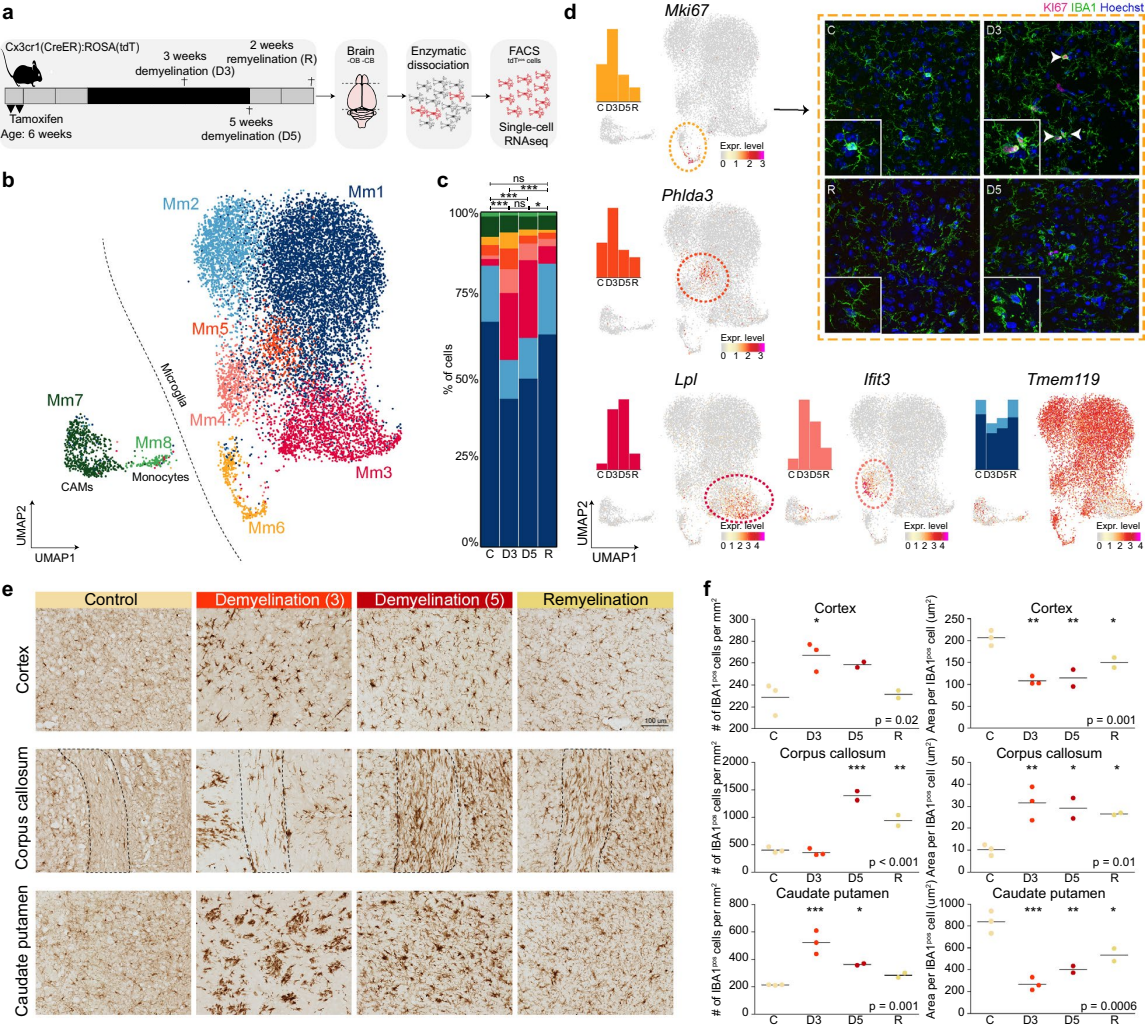
Activated/phagocytic microglia arise during demyelination. **a** Workflow of the experiment. **b** UMAP depicting 12,885 cells. Colors depict unsupervised clustering. **c** Bar plot depicting cluster distribution per group with Chi-squared statistical analysis. **d** UMAPs depicting gene expression levels of *Mki67*, *Phlda3*, *Lpl*, *Ifit3* and *Tmem119*. Immunofluorescent staining of caudate putamen region. Hoechst (blue; nuclei), IBA1 (green; macrophages), Mki67 (magenta; Mm6). Arrowheads indicate IBA1^pos^KI67^pos^ cells. **e** Representative images of IBA1 immunohistochemistry in the cortex, corpus callosum and caudate putamen. **f** Quantification of immunohistochemistry of 2–3 mice per group. *: p < 0.05; **: *p* < 0.01; ***: *p* < 0.001. Abbreviations: C = control; D3 = 3-weeks demyelination; D5 = 5-weeks demyelination; R = remyelination

To validate the hypothesis that Hs7 cells are associated with demyelination, we profiled brain macrophages in the cuprizone mouse model of de- and remyelination. Mice were fed with cuprizone or control diet for 3 or 5 weeks and macrophages were isolated for scRNAseq from the whole brains with exclusion of the olfactory bulb, cerebellum and brainstem (Fig. 4a). Cx3cr1^pos^ cells were FACS sorted (Additional file 4: Fig. S4a) from a Cx3cr1^lox−stop−tdTomato^ reporter mouse strain one month after tamoxifen administration to activate the reporter, and were clustered based on their gene expression profiles (Fig. 4b, Additional file 4: Fig. S4a, b). Significant changes in cluster distribution between the groups were identified (Fig. 4c). Marker genes for each cluster were identified by comparing each cluster with all other cells with DEG analysis (Fig. 4b, Additional file 6: Table S6). Expression of genes specific for CAMs (*F13a1*, *Lyve1*, *Cd163*) and monocytes (*H2-Ab1*, *H2-Aa*, *Cd74*, *Lyz*) was enriched in clusters Mm7 and Mm8, respectively, and these clusters were equally abundant in all groups (Fig. 4c, Additional file 4: Fig. S4c, d). Expression of genes associated with microglia homeostasis, such as *P2ry12* and *Tmem119,* was enriched in clusters Mm1 and Mm2 and depleted in Mm3, Mm4, Mm7 and Mm8 clusters (Fig. 4d, Additional file 4: Fig. S4c, d, Additional file 6: Table S6). Two clusters with an increased abundance in the demyelination groups (D3, D5) were identified: Mm3 and Mm4 (Fig. 4c). Marker genes of Mm3 were *Lpl*, *Apoe*, *Spp1* and *Axl* (Fig. 4d, Additional file 4: Fig. S4c, d), genes previously identified in microglia in several disease models and during development and myelinogenesis [19–21]. Expression of genes associated with the interferon response, such as *Ifit3*, *Stat1* and *Irf7* was enriched in the Mm4 cluster (Fig. 4d, Additional file 4: Fig. S4c, d). A cluster of cells (Mm5) highly expressing *Phlda3* and *Cdkn1a* (Fig. 4d, Additional file 4: Fig. S4d), associated with apoptosis, was identified which was most abundant after 3 weeks demyelination (Fig. 4c). Additionally, a cluster of cells (Mm6) associated with proliferation was identified, as indicated by high expression of *Mki67 and Top2a* (Fig. 4d, Additional file 4: Fig. S4d), that also increased in abundance after 3 weeks demyelination. This finding was supported by immunofluorescent stainings for the proliferation marker Ki67 with IBA1, which revealed proliferating macrophages in the CP of demyelinated mouse brains after 3 weeks cuprizone (Fig. 4d). Remarkably, in the remyelination group (2 weeks after discontinuation of the cuprizone diet), the cluster distribution was very similar to the control condition (Fig. 4c), indicating that the Mm3 and Mm4 phenotypes may be reversible. The identification of the apoptotic (Mm5) and proliferation (Mm6) clusters in the demyelination groups suggests that Mm3 and Mm4 microglia might be (partly) replaced by proliferating microglia.

Changes in microglia proliferation and transcriptomic profiles were confirmed by altered macrophage morphology (Fig. 4e, f right panel) and density (Fig. 4e, f left panel) in situ. Immunohistochemistry for the macrophage marker IBA1 showed that macrophage morphology was extremely affected in the cortex and caudate putamen after 3 weeks on the cuprizone diet (D3) and nearly completely restored after 2 weeks remyelination. In the corpus callosum, macrophage morphology was changed the most after 5 weeks of cuprizone diet (D5), and this effect was still moderately present after 2 weeks remyelination, indicating that not all brain regions are affected at the same time, likely due to differences in myelin content (Fig. 4e, f). Taken together, these data indicate that the activated/phagocytic profile (Mm3) arises during demyelination and disappears after remyelination.

### Activated/phagocytic microglia are present in lesion types with ongoing demyelination

**Fig. 5:**
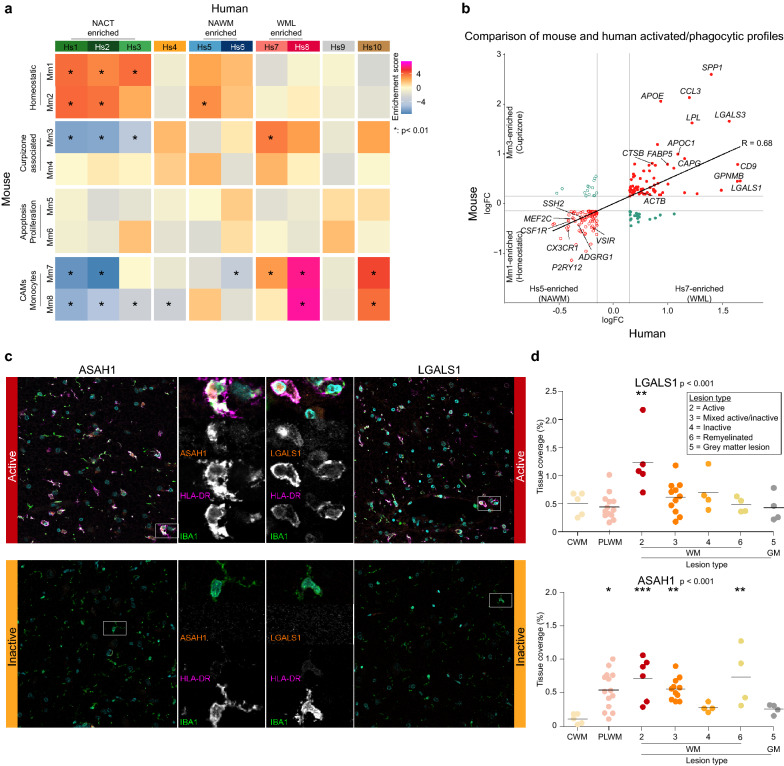
Activated/phagocytic microglia are present in lesion types with ongoing demyelination. **a** Heatmap depicting expression weighted gene set enrichment scores of cluster markers derived from the cuprizone experiment in the human MS dataset. Asterisks indicate significant enrichments (*p* < 0.05). **b** Fourway plot depicting gene signature of human Hs7 cluster compared to Hs5 cluster (x-axis) versus mouse Mm3 versus Mm1 cluster (y-axis). **c** Images of immunofluorescent staining for ASAH1 (orange, left; MS-4), LGALS1 (orange, right; Hs7), HLA-DR (magenta; Hs7), Hoechst (cyan; nuclei) and IBA1 (green; macrophages) in an active and inactive lesion. Scale bar is 25 μm (overview), and 10 μm (zoom). (**d**) Quantification of immunohistochemistry for HLA-DR, LGALS1 and ASAH1 in CTR donors and MS donors amongst 6 different lesion types in 3–11 donors per group. *: *p* < 0.05; **: p < 0.01; ***: *p* < 0.001. Abbreviations: CWM = control white matter (CTR donor); NAWM = normal appearing white matter (MS donor); WML = white matter lesion (MS donor); NACT = normal appearing cortical tissue (MS donor); CAM = CNS-associated macrophages, PLWM = perilesional white matter

Next, we compared the macrophage subtypes identified in the cuprizone mouse study with the MS subtypes. Hs1, Hs2 and Hs3 macrophage clusters showed significant enrichment of marker genes of the Mm1 and Mm2 clusters from the mouse study and depleted expression of Mm3, Mm7 and Mm8 marker genes (Fig. 5a). Expression of Mm7 (CAMs) and Mm8 (monocytes) marker genes was particularly enriched in Hs8 and Hs10 (neutrophils), again showing that the Hs8 cluster may contain CAMs or monocytes rather than microglia. None of the mouse clusters showed significant overlap with the human cluster Hs6, indicating that this is a specific feature of MS pathology that is not recapitulated in the healthy mouse brain or in response to cuprizone-induced demyelination. Conversely, the expression of the mouse apoptotic (Mm5) and proliferating (Mm6) cluster markers was not enriched in any of the human clusters. Expression of the Mm3 marker gene set was significantly enriched in cluster Hs7, and indeed both showed an activated/phagocytic profile (Fig. 5a). Furthermore, cells in clusters Hs7 and Mm3 had comparable differential gene expression profiles compared to homeostatic cells, clusters Hs5 and Mm1 (Fig. 5b). Mm3 microglia arose specifically in relation to demyelination in the cuprizone mouse model (Fig. 4c), therefore we next investigated the association of Hs7 microglia with particular MS lesion types. In order to validate Hs7 microglia in situ, we selected marker genes with a high logFC, that are not expressed in other CNS cell types, not secreted, have published antibodies available and have either been described to play a role in MS (LGALS1), or are novel for MS (ASAH1). Immunohistochemical and immunofluorescent stainings for these two marker genes of cluster Hs7 in five different types of MS lesions in 3–11 independent donors showed that ASAH1^pos^ and LGALS1^pos^ cells (Hs7) were particularly observed in active and mixed active/inactive lesions. These data confirm that Hs7 macrophages with an activated/phagocytic profile are associated with (active) demyelination (Fig. 5c, d, Additional file 5: Fig. S5a, b).

Taken together, this study indicates an early stress response in macrophages in NAWM of MS brains, and that the activated/phagocytic microglia profile in WML is an early but persistent response to disease, associated with ongoing demyelination during lesion formation and progression.

## Discussion

Here, transcriptomic profiling of white matter from control donors, and NAWM and WMLs from MS brain tissues was performed to detect early changes that may underlie MS disease onset. This total tissue transcriptomic profiling pointed towards changes in brain macrophages in NAWM tissue, and associated with stress and inflammatory pathways. Next, we performed scRNAseq of brain macrophages to investigate how these cells are affected in NAWM and WML tissue. We observed a large spectrum of macrophage/microglia heterogeneity in human MS brains, Besides homeostatic microglia, we observed signatures that were nearly exclusively observed in NAWM and WML and displayed disease-associated signatures that overlapped with those found in disease models, during development and in other neurodegenerative diseases [15, 19, 20, 22, 23].

Using scRNAseq on macrophages isolated from post-mortem brain tissues, a cluster of cells was identified that was particularly abundant in NAWM samples and showed enriched expression of heat shock proteins and immediate-early genes (Hs6). These genes have been reported to be induced by ex vivo activation of macrophages, such as incubation steps at 37 °C [24]. Importantly, our cell isolation was performed at 4 °C throughout the entire procedure. This stress-related gene signature was also identified in the total tissue bulk RNAseq dataset, both by us and partly by Melief et al. [8], suggesting that these genes are not induced by cell isolation procedures but are a feature of MS pathology that already occurs in NAWM, which is not demyelinated. Alternatively, the stress signature might represent a particular sensitivity of cells in MS tissue to the processes in the CNS following death that is not observed in macrophages in control donors. Even more, these stress-associated macrophages did not appear in cuprizone-induced demyelinated mice, confirming that the stress macrophage cluster does not arise in response to demyelination itself. These data show that brain macrophages display an early stress response in NAWM, suggesting that these are amongst the first cells to be involved in the onset of demyelination. Alternatively, the stress response in brain macrophages may represent a protective response of these cells, aiming to prevent lesion formation in that tissue area.

Two clusters of macrophages were identified that were particularly enriched in demyelinated WML samples. Cluster Hs8 showed enriched expression of several well-known CAM genes, such as *F13A1* and *LYVE1* [4]. Additionally, cluster Hs8 was significantly enriched for marker gene expression of the CAMs and monocytes that were identified in the cuprizone dataset. The role of microglia and infiltrating macrophages and monocytes in MS brains is complicated by the fact that they are remarkably similar in case of disease [25, 26]. While under homeostatic conditions microglia express certain unique markers, including TMEM119, these microglia markers are downregulated in disease [25, 26]. At the same time, following exposure to the CNS microenvironment of microglia-deficient mice, CNS-infiltrating macrophages adopt a microglia-like phenotype [27]. Therefore, despite the detection of marker genes for CAMs, from these data we cannot definitively conclude whether Hs8 macrophages indeed represent CAMs or that they are microglia that express CAM marker genes in response to pathology. In mice, it was shown that microglia outnumber CAMs and dominate the CNS lesion in response to demyelination [28], making it more likely that these Hs8 macrophages originate from microglia rather than CAMs. On the other hand, blood–brain–barrier (BBB) dysfunction is a well-known feature present in WMLs. BBB dysfunction makes the CNS prone to increased infiltration of macrophages and/or monocytes [29]. In non-active lesions and control brain tissues, Hs8 macrophages were rare and almost exclusively observed near blood vessels. In (pre)-active and chronic lesions, Hs8 macrophages were more frequent and also present in the parenchyma, suggesting that Hs8 macrophages may come from blood vessels and invade the lesion parenchyma, or, alternatively, that resident microglia switch to an Hs8 phenotype.

Cluster Hs7 showed an activated/phagocytic microglia signature that was similar to profiles reported in mouse models of disease [4, 20], human AD [15] and during fetal/neonatal development and myelinogenesis [19, 30, 23]. Hs7 microglia were particularly detected in lesion types with ongoing active demyelination. A similar gene expression profile was identified in the cuprizone dataset (Mm3), supporting their association with active demyelination. Additionally, these Mm3 microglia were depleted when the brain was fully remyelinated. During mouse development, a similar subset of microglia (axon tract associated microglia) appears in regions that become heavily myelinated and disappears when myelination is complete [19]. Another study also showed that a similar microglia subtype (CD11C^pos^) is abundant in areas of primary myelination and is a critical source of IGF1-driven myelination [23]. These data support our notion that in WMLs of MS brains, a phagocytic/activated microglia profile (Hs7) emerges that is similar to those found in the developing brain and is associated with myelin-processes.

Despite promising findings for the field, we are aware of some study limitations. Control donors were not included, which was overcome by processing NACT from the same donors as the NAWM and WML samples. This strategy reduces donor variation. Additionally, MS donors die relatively young, hampering an age-matched design. Furthermore, availability of human post-mortem brain tissue is still very limited. Therefore, all tissue was required for scRNAseq, which made lesion characterization impossible. However, extensive validation with a higher number of well characterized lesions confirmed our findings.

Taken together, our data reveals that macrophages in MS brains adopt a diverse range of phenotypes. Furthermore, significant changes were observed in NAWM tissues indicating that brain macrophages respond to environmental cues, independent of demyelination. These data offer insight into potentially early disease-associated changes in MS brain tissues in relation to lesion development and progression, that may provide therapeutical targets to prevent or reduce demyelination.

## Materials and methods

### Bulk RNAseq experiment

#### Post-mortem frozen human brain tissue and lesion classification

Snap frozen white matter (WM) MS brain tissues were obtained from the Netherlands Brain Bank (NBB, Amsterdam, The Netherlands) (n = 117). From these MS donors the diagnosis progressive MS was confirmed. Age and gender matched post-mortem brain tissues from control donors were obtained from the Edinburgh Brain and Tissue Bank (EBTB, Centre for Clinical Brain Sciences, University of Edinburgh) (n = 35). WM tissue blocks from MS donors and controls were characterized by immunohistochemistry. Per tissue block every first and last two cryosections (5 µm) were used for immunohistochemical stainings, while the intermediate part was used for bulk mRNA sequencing. For sample characterization, the presence or absence of lesions or inflammation was confirmed by scoring demyelination and immune activation through the markers proteolipid protein (PLP1) and Human Leukocyte Antigen – DR isotype (HLA-DR) respectively. An HLA-DR and PLP1 score ranging from 0 to 10 was assigned as previously described [31, 32] (Additional file 1: Fig. S1a). Tissues with PLP1 score 0 (not intact myelin) and HLA-DR score higher than 5 (high macrophage activation) were included in the study as WM lesions, while all tissues with PLP1 score 10 (intact myelin) and HLA-DR score lower than 4 (low macrophage activation) were included in the study as NAWM. In addition, the PLP1 staining was used to determine if the white matter samples were contaminated with grey matter areas. Samples with grey matter contamination were excluded for further analysis.

Cryosections were fixed with acetone (for HLA-DR) or 4% paraformaldehyde (for PLP1) for 10 min and 70% ethanol for 5 min, followed by a 3 min incubation in PBS. Next, endogenous peroxidase activity was suppressed using 0.3% H_2_O_2_, followed by blocking in 5% normal horse serum (NHS) in PBS for 30 min. Sections were incubated overnight with mouse anti-human HLA-DR (eBioscience, 17-9956-42, 1:750) or mouse anti-human PLP1 (Serotec, MCA839G, 1:500), followed by incubation with biotin-conjugated horse-anti mouse secondary antibody (Vector, BA-2000-1.5, 1:400) for 2 h at room temperature. After a 30 min incubation with the avaidin-biotin solution (ABC, Vectastain ABC kit, Vector, PK-6100), the complex was visualized with DAB in PBS containing 0.03% H_2_O_2_. Subsequently, haematoxylin was used as nuclear counterstain, followed by mounting in DePeX (Serva, 18243). Images were digitalized using a NanoZoomer 2.0-HT Digital slide scanner C9600 (Hamamatsu Photonics). Sections were scored for HLA-DR and PLP1 using an Axioskop microscope (Carl Zeiss).

#### Bulk RNAseq of frozen brain tissues

WM snap frozen tissue blocks were classified as WML containing either an active, mixed active/inactive or remyelinated lesion (for lesion classifications of each sample based on Luchetti et al. 2018 [16] see Additional file 6: Table S1), NAWM and CWM (Additional file 1: Fig. S1a). Tissue sections immediately surrounded by the sections used for classification were collected for RNA extraction and whole tissue 3**’** mRNAseq. This concerned 20 10 µm cryosections that were collected in 1 mL QIAzol (Qiagen, 79306) and lysed and homogenized with a syringe. RNA was isolated using the RNeasy Lipid Tissue mini kit (Qiagen, 74804) following manufacturer’s instructions. RNA was eluted in 40 µl RNase free water, RNA concentrations and integrity were measured on a Bioanalyzer 2100 (Aligent). Samples with a RIN value > 4 were included for downstream analysis. cDNA libraries were generated with the QuantSeq 3’ mRNA-Seq Library Prep Kit FWD for Illumina (Lexogen, 01596) according to the manufacturer’s protocol. Samples were sequenced on an Illumina NextSeq 500 Sequencing System with NextSeq 500/550 High Output Kit v2.5 (Illumina, 20024906).

#### Bulk RNAseq analysis

For bulk RNAseq analysis, raw reads were aligned to the GRCh37 reference genome from Ensemble with Hisat (v0.1.5). Aligned reads were sorted with samtools (v1.2) and counted with HTSeq (v0.6.1). FastQC (v0.11.3) and Picard (v1.130) were used to perform quality control. Raw count matrices were loaded in R and annotated by converting the ensemble IDs to gene symbols using the corresponding.gtf file. Lowly expressed genes were filtered using a data-adaptive flag method for RNA-sequencing (DAFS [33]). Only genes with > 1 counts in at least 2 samples were included in the analysis. To determine whether donors from the same group would cluster together, the count matrix was normalized with the blinded variance-stabilizing method from DESeq2 from Bioconductor and mitochondrial genes were removed prior to this analysis. A negative binomial generalized log-linear model was used to model gene expression levels and differentially expressed genes were determined using a likelihood ratio test [34]. Thresholds were set at abs(logFC) > 1 and adjusted-*p* < 0.05. Principal component analysis was performed on VST-transformed counts. Visualizations were made with the CRAN package ‘ggplot2’. For WGCNA analysis, VST-transformed counts obtained from DESeq2 were used as input [35, 36]. Signed WGCNA was performed using biweight mid-correlations and the maximum number of excluded outliers was restricted to 10% [37]. Gene ontology analysis was performed with MetaScape [38]. Cell (sub)type enrichment analyses were performed with expression weighted cell type enrichment analysis (EWCE) [13] using CTR donors from the snRNAseq dataset from [15] as a reference. For DEG gene-sets, logFC was used for gene ranking in EWCE, for WGCNA the module member ship scores.

### Human brain macrophage experiment

#### Fresh post-mortem human MS brain tissue

Of 5 donors, fresh post-mortem brain tissue was obtained from the Netherlands Brain Bank (NBB). Immediately after autopsy, the tissue was transported from Amsterdam to Groningen in HBSS with phenol red (Thermofisher Scientific, 14170-088) supplemented with 15 mM HEPES (Lonza via Westburg, LOBE17-737E) and 0.6% glucose (Sigma Aldrich, G8769). Of each donor, three samples were obtained: i.e. (1) normal-appearing cortical tissue mainly consisting of a mixture of grey- and white matter (NACT); (2) normal-appearing white-matter (NAWM); (3) white-matter lesion (WML) tissue with the surrounding perilesional WM area (Fig. 3a). Lesions were selected by the NBB based on post-mortem magnetic resonance imaging (MRI) together with detailed macroscopic observations as described on their webpage. Due to limited availability of fresh post-mortem MS tissues and the relatively small size of the tissue samples with MS lesions, all obtained tissue was used for single cell sequencing and, therefore, the MS lesion type and HLA-DR activity within the NAWM are unknown. Furthermore, due to limited control donors of similar ages, tissue from aged matched control donors was not available. To correct for age as a batch effect and to allow for within donor comparisons and generate sufficient contrasting signatures to identify disease-associated signatures, we included multiple samples from each donor. Besides WML and NAWM tissue, NACT was used as an internal reference group, since pathology in GM is known to be distinct from WM pathology. Informed consent to perform autopsies and the use of tissue and clinical data for research purpose were obtained from donors and approved by the Ethical Committee of the VU University Medical Center (VUmc, Amsterdam, The Netherlands).

#### Macrophage isolation from human brain tissue

Human macrophages were isolated as described previously [14]. In brief, meninges were removed and the tissue was mechanically dissociated using a glass tissue homogenizer. A cell suspension was obtained via filtering through 300-µm and 106-µm sieves. Myelin was removed by a 24% Percoll gradient (Fisher Scientific, 17-0891-01) in 10 × HBSS (Gibco, 14180-046) and phosphate buffered saline (PBS) density gradient centrifugation and followed by a second centrifugation step containing a 60% and 30% Percoll layer with PBS on top. The interphase between the Percoll layers was collected and contained the immune cells. Fc receptors were blocked with human Fc receptor-binding inhibitor (eBioscience, 14-9161-73). For FACS, cells were incubated for 20 min with anti-human CD11B-PE (BioLegend, 301306) and anti-human CD45-FITC (BioLegend, 304006) and washed with HBSS without phenol red (Thermofisher Scientific, 14175-053). The cells were passed through a 35-μm nylon mesh, collected in round bottom tubes (Corning 352235), stained with DAPI (Biolegend, 422801) and DRAQ5 (Thermofisher Scientific, 62251) and sorted using a Beckman Coulter MoFloAstrios, Beckman Coulter MoFloXDP or Sony SH800S cell sorter. Human macrophages were sorted as DAPI^neg^DRAQ5^pos^CD11B^pos^CD45^pos^ (Additional file 2: Fig S2a).

#### Single-cell RNA sequencing mouse and human cells

The single cell cDNA libraries were constructed using the Chromium Single Cell 3’ Reagents Kit v2 and corresponding user guide (10 × Genomics) and sequenced on a NextSeq 500 at the sequencing facility in the UMCG up to a depth of ~ 20,000 reads/cell.

#### scRNAseq data analysis

Raw reads were aligned to the GRCh38 or GRCm38 genome for human and mouse samples, respectively, using Cell ranger (v3.0.0) with default settings. Raw count files were loaded into R (v3.6) and barcode filtering was performed with thresholds at > 600 unique molecular identifiers (UMIs) for mouse cells and > 400 for human cells [39]. The multiplet rate mentioned in the 10 × Genomics User Guide was used to set an upper threshold per sample for the number of UMIs per cell. Cells with a mitochondrial content > 5% were removed from the dataset.

For the mouse dataset, the count files from different conditions were merged into one and further analyzed with Seurat [40]. The data were normalized by dividing the counts of each gene by the total sum of counts per cell and multiplied by a scale factor of 10,000 and log-transformed. Highly variable genes (HVGs) were calculated using the ‘VST’ method with default settings. The data was scaled and heterogeneity associated with number of UMIs and mitochondrial and ribosomal content were regressed out, then the data was clustered. Differential gene expression analysis was performed with MAST [41].

For the human dataset, count matrices of the three brain regions per donor were merged into one file per donor and normalized using the same method as for the mouse data. HVGs were determined using the VST method. The datasets from the donors were integrated using canonical correlation analysis [40]. The data was scaled and heterogeneity associated with number of UMIs, mitochondrial content, sex and ribosomal content were regressed out. The data was clustered using the graph-based clustering method implemented in Seurat with default settings. Differential gene expression was performed on the unintegrated data using logistic regression with donor as a latent variable. Geneset module scoring was performed using the ‘AddModuleScore’ function in Seurat. Cell (sub)type enrichment analyses were performed with EWCE using the human scRNAseq as a reference dataset and marker genes of the cuprizone scRNAseq clusters ranked by logFC [13]. Statistical analysis of cluster distribution changes between groups was performed using chi-squared tests in R.

#### Gene sets from literature

From [22], EV7 was downloaded and genes with a p_val_adj < 0.05 and abs(logFC) > 0.15 were selected. From [19], Additional file 6: Table S1 was downloaded and marker genes from cluster 4 were selected. From [20], Additional file 6: Table S2 was downloaded and upregulated genes of “Microglia3” with a p_val_adj < 0.05 were selected. From [23], dataset EV1 was downloaded and DEGs between neonatal CD11c^pos^ microglia and neonatal microglia with abs(logFC) > 2.5 and FDR < 0.05 were used for Fig. 3g. DEGs between each cluster compared to all other cells were used for Additional file 3: Fig. S3c (p_val_adj < 0.05). From [15], DEGs between AD1 microglia and homeostatic microglia (abs(logFC) > 0.15 and p_val_adj < 0.05) were used. From [30] cluster markers of cluster 8 were used (abs(avg_logFC) > 0.25 and p_val_adj < 0.05).

### Paraffin-embedded human brain tissue

Formalin-fixed paraffin-embedded, well-characterized tissues from 5 controls and 14 MS donors were obtained from the NBB. Lesions were classified based on HLA-DR and PLP1 immunohistochemistry according to the system for lesion classification from the NBB [16], resulting in the following lesion types indicated by a number: active (2), mixed active/inactive (chronic) (3), inactive (4) and shadow plaques or remyelinated lesions localized in WM (6) and GM lesions (5). Paraffin blocks were cut into 6-µm thick sections. After deparaffinization and heat-induced antigen retrieval in 10 mM sodium citrate (pH 6.0) with 0.05% Tween for 10 min in a microwave, sections were divided in two series. We used one series of sections for immunohistochemistry and subsequent quantification and MS lesion scoring, using the Avidin–Biotin Complex (ABC) method followed by a DAB staining. The other series of sections we used for triple immunofluorescence stainings with various antibody combinations.

#### Immunohistochemistry

After deparaffinization and antigen retrieval, endogenous peroxidase was blocked using H_2_O_2_/PBS 0.3% for 30 min. After washing with PBS, tissue sections were incubated for 30 min with 2% normal serum (NS) and 2% bovine serum albumin (BSA). After a short wash with PBS, sections were incubated overnight at 4 °C with the primary antibody in PBS containing 1% NS and 1% BSA (Table 1). The following primary antibodies were used (Table 1): anti-PLP1 (AbD Serotec, MCA839G, dilution 1:100), anti-HLA-DR (eBioscience, 14-9956, dilution 1:500), anti-FCGR2B (Bioorbyt, orb44658, dilution 1:350), anti-ASAH1 (Abcam, ab74469, dilution 1:500), anti-LGALS1 [42, 43] (dilution 1:100). Next day, sections were washed with PBS and incubated for 2 h with the appropriate biotinylated secondary antibodies (anti mouse or rabbit, Vector Labs, dilution 1:400; Table 1), followed by a 30 min incubation with the ABC solution (Vectastain elite kit, PK-6100). After rinsing with PBS, the sections were incubated for 10 min in DAB and 0.03% H_2_O_2_. Finally, the sections were counterstained with cresyl violet, dehydrated, and mounted with DepeX (Serva, 18243). Images were acquired using a NanoZoomer 2.0-HT Digital slide scanner C9600 (Hamamatsu Photonics). Images of the HLA-DR and PLP1 stainings were used for scoring the MS lesion, the results of the FCGR2B, ASAH1 and LGALS1 stainings were quantified as follows. From every type of lesion five 40× magnified images were extracted per donor. To measure FCGR2B activity near blood vessels, 80X magnified images were extracted from the scans. Analysis was performed using FIJI by first applying colour deconvolution using the H & DAB method. From the DAB channel a binary image was made using manual thresholding and the area fraction was measured in ImageJ. Statistics were performed with a two-way ANOVA for location and lesion type, followed by Tukey post-hoc test for lesion types (Fig. 3i, j). Statistics on DAB total tissue coverage quantifications were performed with a one-way ANOVA followed by Dunnet’s test for comparison of each group versus the CWM group (Fig. 5d).

**Table 1:** Antibodies used for IHC and FACS

Immunohistochemistry			
	Company	Cat #	Dilution
*Antibody (primary)*			
PLP1 (clone plpc1)	AbD Serotec	MCA839G	1:500
HLA-DR (clone LN3)	eBioscience	14-9956	1:750
FCGR2B (datasheet CD32B)	Biorbyt	orb44658	1:350
ASAH1	Abcam	ab74469	1:500
LGALS1			1:100
Claudin5 (clone 4C3C2)	Thermo Fisher Scientific	35-2500	1:250
IBA1	Wako	019-19,741	1:1000
IBA1	Abcam	ab5076	1:750
Ki67 (Clone B56)	BD Pharmingen	556,003	1:400
*Antibody (secondary)*			
Biotin-Donkey anti-Rabbit IgG	JacksonImmunoResearch	711-065-152	1:300
Cy3 Goat anti-Mouse IgG	JacksonImmunoResearch	115-165-003	1:200
Donkey anti-Mouse, Alexa Fluor™ 594	Thermo Fisher Scientific	A21203	1:300
Donkey anti-Goat, Alexa Fluor™ 633	Thermo Fisher Scientific	A21082	1:300
Streptavidin, Alexa Fluor™ 488	Thermo Fisher Scientific	S11223	1:300

FACS			
Antibody (primary)	Company	Cat #	Dilution
FITC anti-human CD45	BioLegend	304,006	1:25
PE anti-human CD11b	BioLegend	301,306	1:40

#### Immunofluorescence

After deparaffinization and antigen retrieval as described above, sections were incubated for 10 min in a Sudan Black solution (0.3% Sudan Black/Ethanol 70%) to quench autofluorescence followed by dipping the slides 3 times in ethanol 70% to remove excess of Sudan Black. After washing with PBS, tissue sections were incubated for 30 min with 2% NS and 2% BSA. After a short wash with PBS, sections were incubated overnight at 4 °C with primary antibodies in PBS containing 1% NS and 1% BSA (Table 1). A mixture of goat anti-Iba1 (Abcam, ab5076, dilution 1:500) and mouse anti-HLA-DR (eBioscience, 14-9956, dilution 1:500) or mouse anti-CLDN5 (Thermo Fisher Scientific, 35-2500, 1:250) was in various separate experiments supplemented with antibodies raised in rabbit, namely anti-FCGR2B (Bioorbyt, orb44658, dilution 1:350), anti-ASAH1 (Abcam, ab74469, dilution 1:500), anti-LGALS1 [42, 43] (dilution 1:100). After an overnight incubation at 4ºC, the sections were rinsed and incubated with a biotinylated anti-rabbit antibody (anti-rabbit, Vector Labs, dilution 1:400), followed by an incubation of 1 h at room temperature with donkey anti-mouse IgG (H + L) AlexaFluor™ 594 (Thermo Fisher Scientific A21203), donkey anti-goat IgG (H + L) AlexaFluor™ 633 (Thermo Fisher Scientific A21082) and Streptavidin, AlexaFluor™ 488 conjugate (Thermo Fisher Scientific S11223) all diluted 1:300 in PBS plus Hoechst stain (Sigma 14530, 5 μM final concentration). Sections were washed with PBS and demi water, mounted with Mowiol and imaged on a Leica SP8X confocal laser scanning microscope (Leica Microsystems, Amsterdam).

### Animal experiment

#### Cuprizone mouse model

C57BL/6J-Cx3cr1^tm2.1(cre/ERT2)Litt^Gt(ROSA)26Sor^tm14(CAG-tdTomato)Hze^ mice were used in a model for cuprizone-induced demyelination. Genotype for the experimental animals was confirmed by genomic PCR for Cx3cr1 and Tomato alleles. Genomic DNA was extracted from earcuts of mice using MyTaq Extract-PCR kit (Bioline, BIO-21127) according to manufacturer’s instructions. The alleles were amplified using MyTaq HS Red mix (Bioline, BIO-25047) and analysed on a 1,5% agarose gel. For Cx3cr1 WT and creERT2 alleles, two primer-pairs were used (WT_fwd: 5′-CTCAC GTGGA CCTGC TTACTG; WT_rev: 5′-GTACC GGTCG ATGCT GATGA and creERT2_fwd: 5′-AAGAC TCACG TGGAC CTGCT; creERT2_rev: 5′-CGGTT ATTCA ACTTG CACCA) with the following PCR program: (1) 95 °C 3 min, (2) 95 °C 15 s, (3) 58 °C 15 s, (4) 72 °C 20 s, repeated 30 times. Cx3cr1 WT alleles present with a band at 482 bp, whereas Cx3cr1 creERT2 alleles present with a band at 260 bp. For Rosa WT and Tomato alleles two primer-pairs were used. For the WT allele (fwd: 5’-AAGGGAGCTGCAGTGGAGTA; rev 5’-CCGAAAATCTGTGGGAAGTC) and Tomato allele (fwd: 5’-CTGTTCCTGTACGGCATGG; rev 5’-GGCATTAAAGCAGCGTATCC) with the following PCR program: (1) 95 °C 3 min, (2) 95 °C 15 s, (3) 68 °C 15 s (decrease 1C/cycle), (4) 72 °C 20 s, repeated 9 times,: (5) 95 °C 15 s, (6) 58 °C 15 s, (7) 72 °C 20 s, repeated 24 times. Rosa WT alleles present with a band at 297 bp, whereas Rosa Tomato alleles present with a band 196 bp. The mice were bred in-house on a C57BL/6 J background. In order to activate Cre-recombinase and express the tomato reporter in CX3CR1 expressing cells, 6-weeks-old animals received twice 500 mg/kg body weight tamoxifen (Sigma Aldrich, T5648-5G) dissolved in corn oil (Sigma Aldrich, C8267-500ML) via oral gavage with a 3-day interval. All animal procedures were approved by the local central authority for scientific procedures on animals (CCD) and performed in accordance to ethical regulations (AVD105002015360). Demyelination was induced in 8-week-old male mice via a diet containing 0.2% w/w cuprizone (Sigma Aldrich, C9012-25G). The chow diet was freshly prepared every week by mixing cuprizone with standard powder food and water, and stored at − 20 °C. Animals were provided with this home-made chow three times a week ad libitum. Control animals received similarly prepared chow, but without cuprizone. The experimental timepoints were: early demyelination (3 weeks cuprizone diet), complete demyelination, start remyelination (5 weeks cuprizone diet) and remyelination following withdrawal of the cuprizone diet for 2 weeks. To reduce biological variation and limit the effect of individual animals on the data, macrophages from n = 5 animals per group were pooled into one sample for scRNAseq. Additionally, tissues from 3 animals per group were collected for immunohistochemistry.

#### Macrophage isolation from mouse brain tissue

Mice were sacrificed under deep anaesthesia (4% isoflurane with 7.5% O_2_) and perfused with cold PBS. After perfusion, brains were removed from the skull and kept in cold medium A (HBSS (Gibco, 14170-088) with 0.6% glucose (Sigma, G8769) and 7.5 mM HEPES (Lonza, BE17-737E)). Macrophages were isolated enzymatically from the whole brain minus olfactory bulb and cerebellum. Brains were minced on a glass slide into small pieces with a knife and transferred to a tube containing enzyme solution with: 2 mL PBS, 20 mg Protease from *Bacillus licheniformis* (Sigma Aldrich, P5380-1G) and 20 µL L-cysteine, incubated on ice for 15 min while mixing every 5 min. After enzymatic dissociation, the cell suspension was passed through a 100 µm cell strainer (Corning, 21008-950), filled up with 15 mL enriched HBSS. Cells were pelleted by centrifugation for 10 min, 300 RCF at 4 °C. Myelin was removed by 24% Percoll- (Fisher Scientific, 17-0891-01) and PBS density gradient centrifugation for 20 min, 950 RCF at 4 °C. The cells were passed through a 35-μm nylon mesh, collected in round bottom tubes (Corning, 352235) and sorted using a Beckman Coulter MoFloAstrios cell sorter. DAPI^neg^tdTomatoRed^pos^ cells were collected for scRNAseq (Additional file 4: Fig. S4a).

### Immunohistochemistry and immunofluorescence on mouse brain tissue

4% PFA-fixed frozen mouse brains were sectioned at a thickness of 16 µm at bregma − 0.8. This region was selected because most cuprizone induced demyelination is expected between bregma − 0.6 and − 1 based on previous experience. Sections were stained using immunohistochemistry or immunofluorescence method. For both methods, heat-induced antigen retrieval with sodium citrate (pH 6) was applied to unmask the epitopes.

#### Immunohistochemistry

After heat induced antigen retrieval as described above, sections were washed with PBS and incubated with 0.3% H_2_O_2_/PBS to block endogenous peroxidases for 30 min. Blocking was performed for 1 h using 5% normal goat serum diluted in PBS with 0.3% Triton X-100. After washing with PBS, sections were incubated overnight at 4 °C with the primary antibody: IBA1 (Wako, 019-19741, dilution 1:1000) in PBS containing 1% normal goat serum and 0.3% Triton X-100. The next day sections were washed with PBS and incubated with a biotinylated secondary antibody: goat-anti-rabbit (Vector, BA1000, dilution 1:400), followed by a 30 min incubation with the ABC solution (Vectastain elite kit, PK-6100). After rinsing with PBS, the sections were incubated for 10 min in DAB and 0.03% H_2_O_2_. Finally, the sections were dehydrated, and mounted with DepeX (Serva, 18,243). Images were acquired using a NanoZoomer 2.0-HT Digital slide scanner C9600 (Hamamatsu Photonics). Per sample 10X zoomed images were saved from the striatum (1 image), corpus callosum (1 image) and cortex (2 images). For quantification, areas were used and averaged per animal with sizes of twice 500 × 500 µm (striatum), 200 × 625 µm (corpus callosum) and four times 400 × 400 µm (cortex) respectively. IBA1^pos^ cells within these drawn areas were manually quantified using the FIJI plugin ‘cell counter’. Additionally, images were converted to grayscale (8-bit images) and the percentage of IBA1 positive pixels was measured by FIJI. A constant threshold of 160 was used for the quantification. Statistics were performed with a one-way ANOVA followed by Dunnet’s test for comparison of each group versus the control group.

#### Immunofluorescence

After heat-induced antigen retrieval as described above, blocking was performed for 1 h using 5% normal goat and normal horse serum. Sections were incubated overnight with the primary antibodies: Ki67 (BD Pharmingen, 556003, dilution 1:400) and IBA1 (Wako, 019-19741, dilution 1:1000) in order to visualize proliferating microglia. After incubation and washing with PBS tissues were incubated with a secondary antibody mix containing donkey anti-rabbit AF488 (Thermofisher Scientific, A21206, dilution 1:400) and goat anti-mouse IgG Cy™3 (Jackson ImmunoResearch, 115-165-003, dilution 1:200) for 1.5 h. Additionally, nuclei were visualized by Hoechst. Images were acquired with a Leica SP8X confocal laser scanning microscope (Leica Microsystems, Amsterdam) using a HC PL APO CS2 40x/1.30 oil objective in a sequential order with optimized emission using a white light laser and excitation detection using gated HyD detectors.

## Supplementary Information

**Additional file 1**:** Figure S1**. Supplementary data related to figures 1 and 2. (**a**) Representative images of DAB staining for PLP and HLA-DR that was used for sample scoring. (**b**) Heatmap depicting gene expression of a manually chosen set of immediate-early genes. Rows and columns are ordered by hierarchical clustering. (**c**) Box plots depicting sum of IEG expression (top), HSPA1A expression level (middle) and SOCS3 expression level (bottom). (**d**) Box plots depicting module eigengenes per sample group derived from the WGCNA on 68 white matter brain tissue samples. (**e**) Bar plots depicting gene ontology analysis of magenta and pink modules. *:p <0.01; **:p < 0.001; ***: p < 0.0001. Abbreviations: CWM = control white matter (CTR donors); NAWM = normal appearing white matter (MS donors); WML = white matter lesion (MS donors); IEGs = immediate early genes.

**Additional file 2**:** Figure S2**. Supplementary data related to figure 3. (**a**) Representative plots depicting FACS strategy to obtain live CD45posCD11Bpos cells. (**b**) Boxplots per sample depicting several indicated quality control parameters per cell. (**c**) Stacked bar plots depicting cluster distribution per sample with statistical analysis (Chi-squared test). C = NACT; W = NAWM; L = WML. (**d**) Volcano plot depicting significantly differentially expressed genes between NAWM and NACT samples (paired design). (**e**) Bar plots depicting top 15 gene ontology terms associated with DEGs between NACT and NAWM cells. *: p < 0.05; **: p < 0.01; ***: p <0.001. Abbreviations: NACT = normal appearing cortical tissue; NAWM = normal appearing white matter; WML = white matter lesion.

**Additional file 3**:** Figure S3**. Supplementary data related to figure 3. (**a**) Volcano plot depicting significantly differentially expressed genes between Hs8 and Hs6 samples. (**b**) Bar plots depicting top 20 gene ontology terms associated with DEGs between Hs8 and Hs6 cells. (**c**) Heatmap depicting expression weighted gene set enrichment scores of microglia subcluster markers derived from Gerrits et al. (2021) in the human MS dataset. Asterisks depict significant enrichments (p < 0.05). Abbreviations: NACT = normal appearing cortical tissue; NAWM = normal appearing white matter; WML = white matter lesion.

**Additional file 4**:** Figure S4**. Supplementary data related to figure 4. (**a**) Representative plots depicting FACS strategy to obtain live Cx3cr1pos cells. (**b**) Boxplots per sample depicting several QC indicated stats per cell. (**c**) Dot plot depicting expression of mouse homologs of the genes depicted in Figure 3f. (**d**) Dot plot depicting expression of marker genes of each mouse scRNAseq cluster. Size of the symbols depicts the fraction of cells expression the gene, color scale depicts average expression level. Abbreviations: CAM = CNS-associated macrophages.

**Additional file 5**:** Figure S5**. Supplementary data related to figure 5. Representative images of immunohistochemistry for HLA-DR, LGALS1, ASAH1 and FCGR2B in CTR donors and 6 lesion types in MS donors. Abbreviations: CWM = control white matter (CTR donor), PLWM = perilesional white matter.

**Additional file 6**: **Table S1**: Donor and sample information. **Table S2**: Differential gene expression analysis total tissue bulk data. **Table S3**: WGCNA modules. **Table S4**: Differential gene expression analysis between clusters (human single cell data). **Table S5**: Differential gene expression analysis between NAWM and NACT in human single cell data. **Table S6**: Differential gene expression analysis between clusters (mouse single cell data).

## Data Availability

The datasets supporting the conclusions of this article are available through Gene Expression Omnibus at https://www.ncbi.nlm.nih.gov/geo with accession number GSE179427.
