# Supplementary figures and images for: Brain macrophages acquire distinct transcriptomes in multiple sclerosis lesions and normal appearing white matter
Source: Acta Neuropathol Commun. 2022 Jan 28;10:8. doi: 10.1186/s40478-021-01306-3 (PMC8796391; doi:10.1186/s40478-021-01306-3)

a

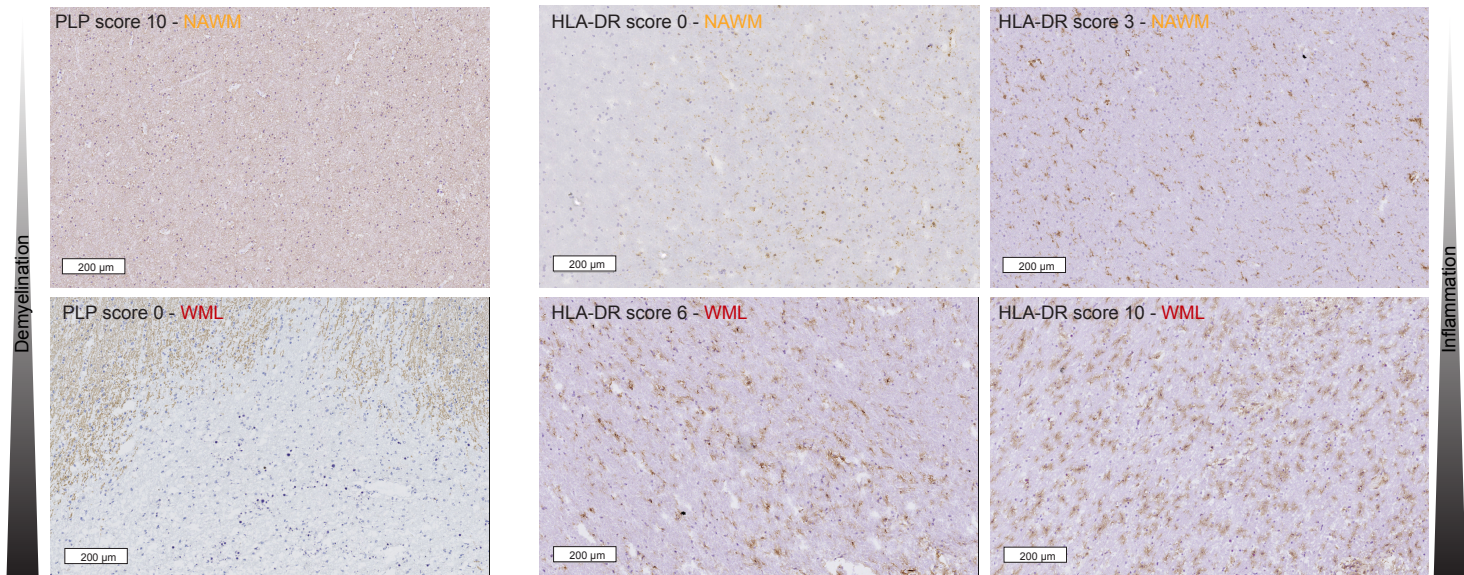

b

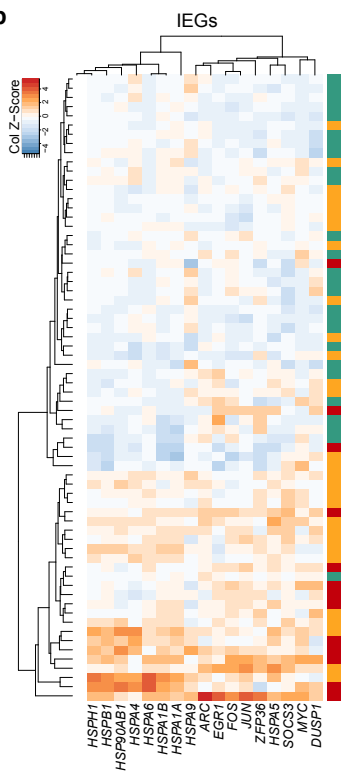

c

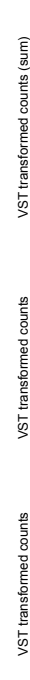

d

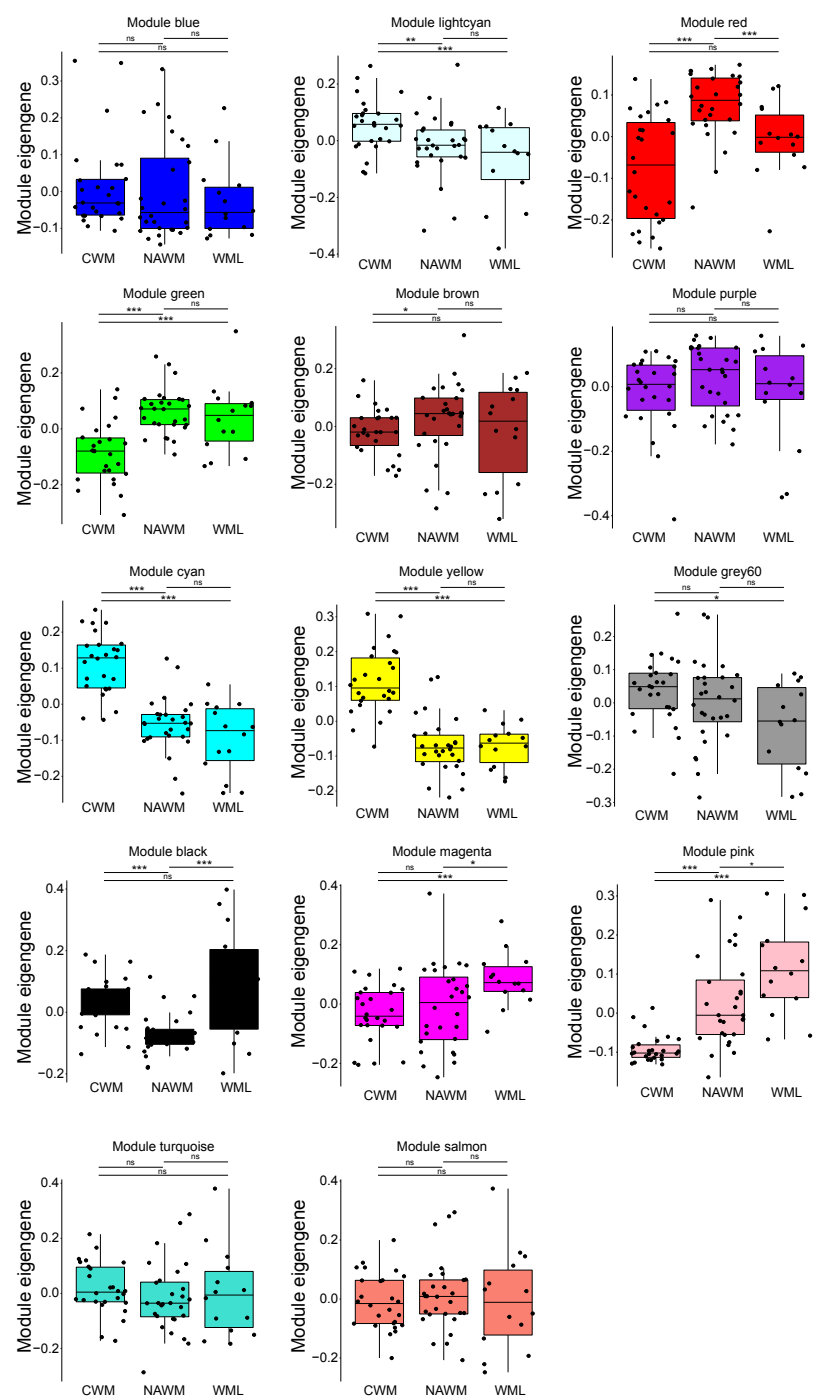

e

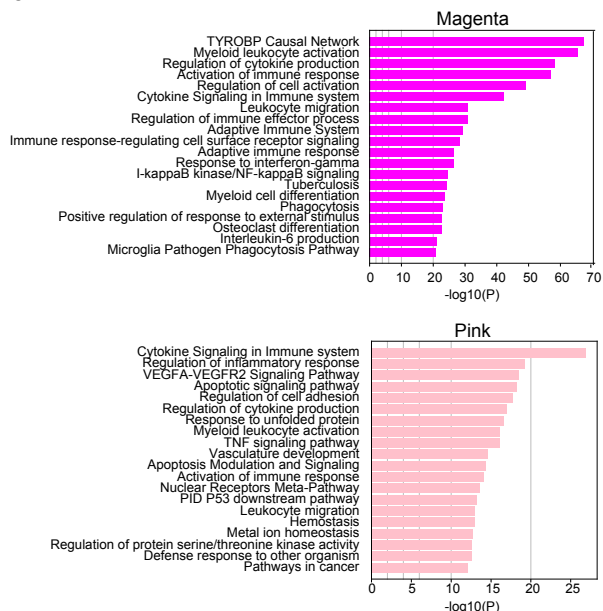

Supplement: Supplementary file 1 — Additional file 1: Figure S1. Supplementary data related to figures 1 and 2. (a) Representative images of DAB staining for PLP and HLA-DR that was used for sample scoring. (b) Heatmap depicting gene expression of a manually chosen set of immediate-early genes. Rows and columns are ordered by hierarchical clustering. (c) Box plots depicting sum of IEG expression (top), HSPA1A expression level (middle) and SOCS3 expression level (bottom). (d) Box plots depicting module eigengenes per sample group derived from the WGCNA on 68 white matter brain tissue samples. (e) Bar plots depicting gene ontology analysis of magenta and pink modules. *:p <0.01; **:p < 0.001; ***: p < 0.0001. Abbreviations: CWM = control white matter (CTR donors); NAWM = normal appearing white matter (MS donors); WML = white matter lesion (MS donors); IEGs = immediate early genes. [file 40478_2021_1306_MOESM1_ESM.pdf]

Supplementary figure S2

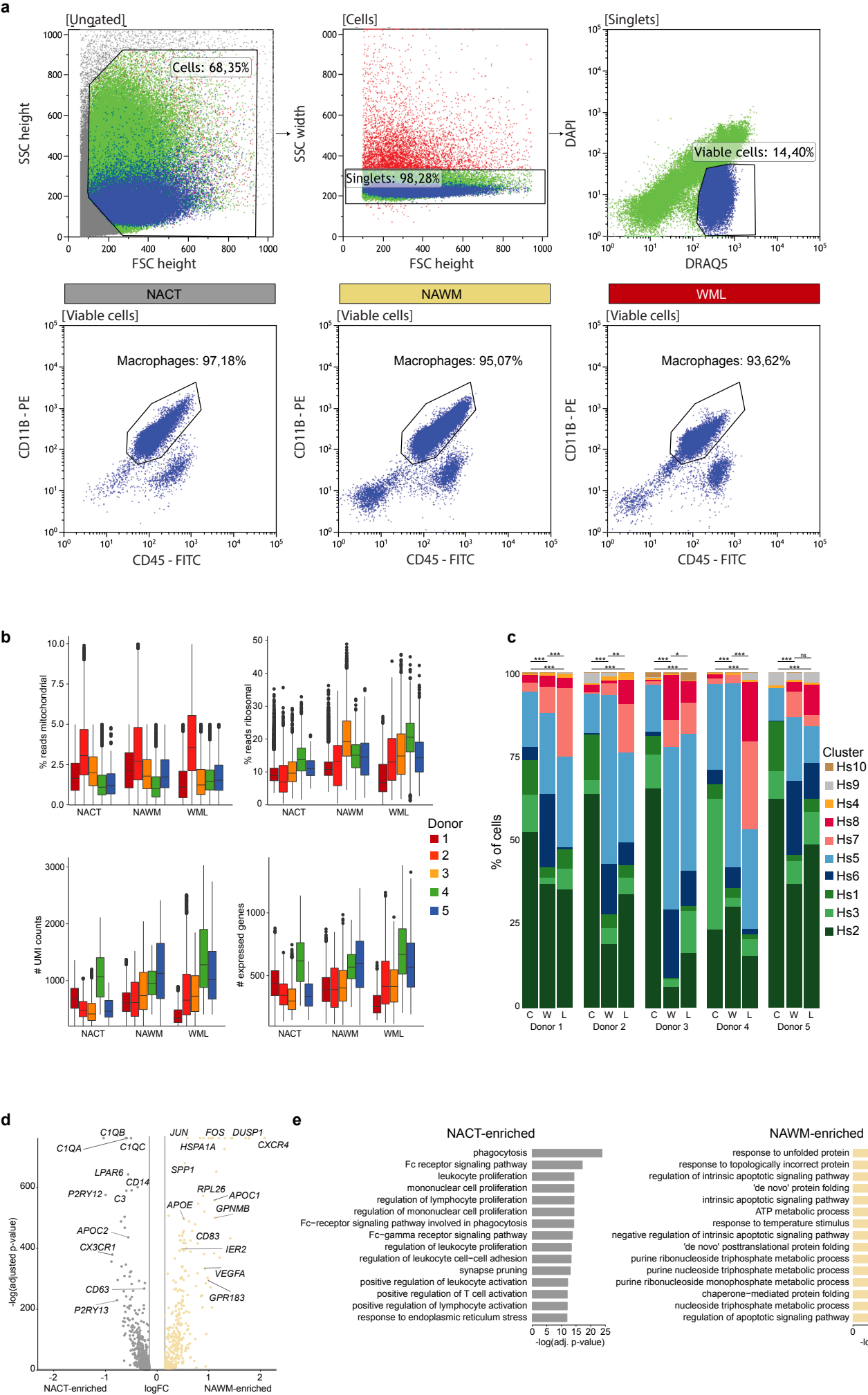

Supplement: Supplementary file 2 — Additional file 2: Figure S2. Supplementary data related to figure 3. (a) Representative plots depicting FACS strategy to obtain live CD45posCD11Bpos cells. (b) Boxplots per sample depicting several indicated quality control parameters per cell. (c) Stacked bar plots depicting cluster distribution per sample with statistical analysis (Chi-squared test). C = NACT; W = NAWM; L = WML. (d) Volcano plot depicting significantly differentially expressed genes between NAWM and NACT samples (paired design). (e) Bar plots depicting top 15 gene ontology terms associated with DEGs between NACT and NAWM cells. *: p < 0.05; **: p < 0.01; ***: p <0.001. Abbreviations: NACT = normal appearing cortical tissue; NAWM = normal appearing white matter; WML = white matter lesion. [file 40478_2021_1306_MOESM2_ESM.pdf]

Supplementary figure S4

**a**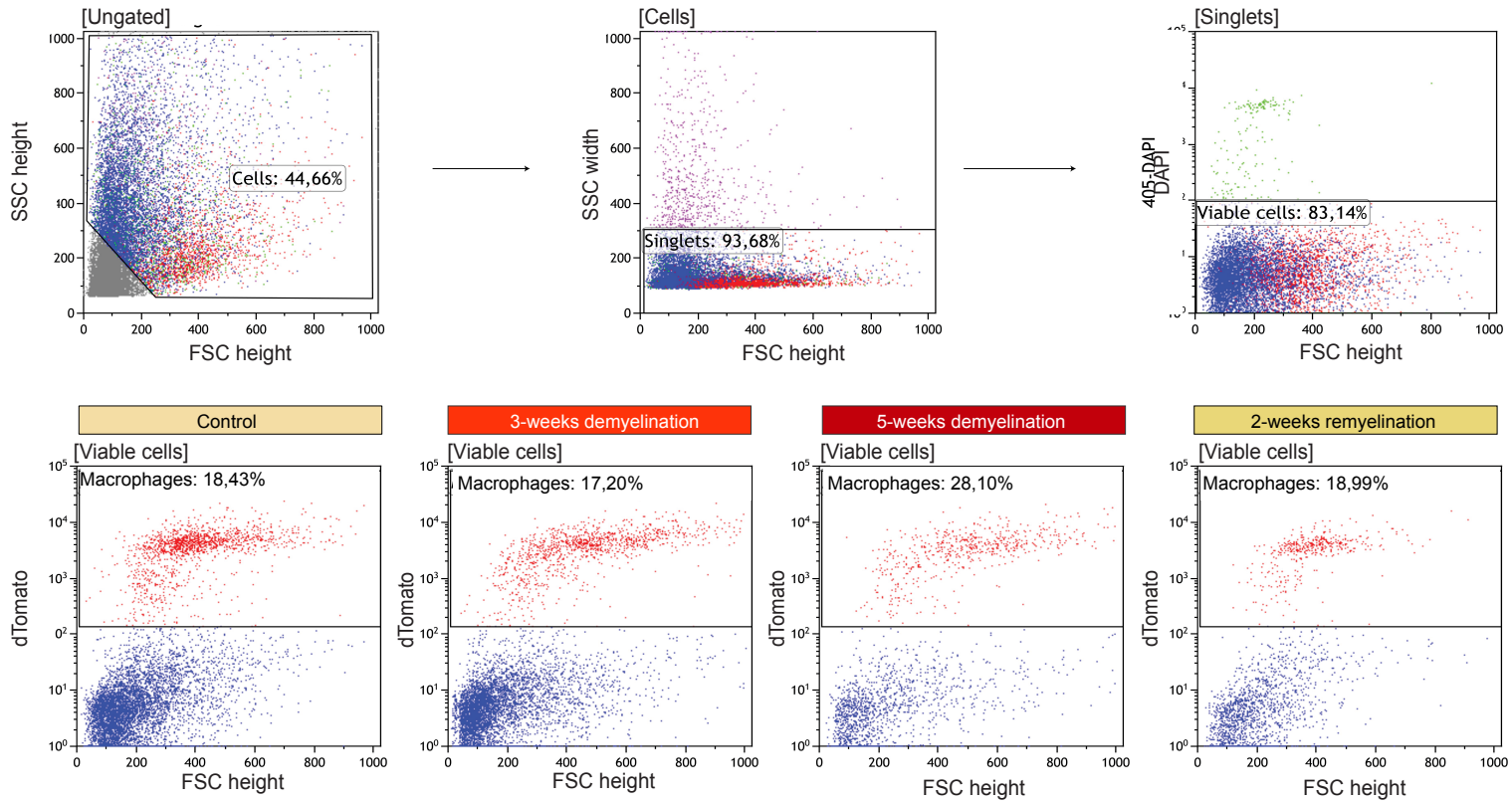**b**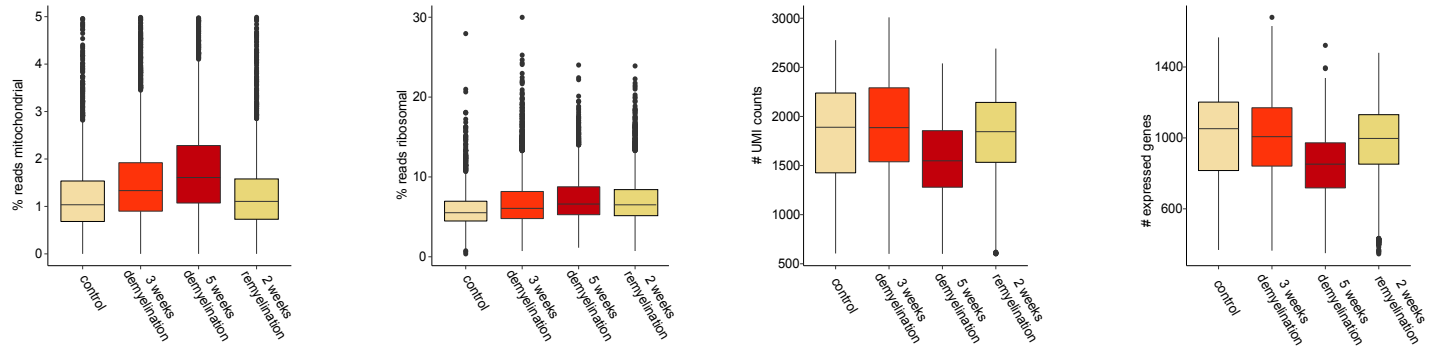**c**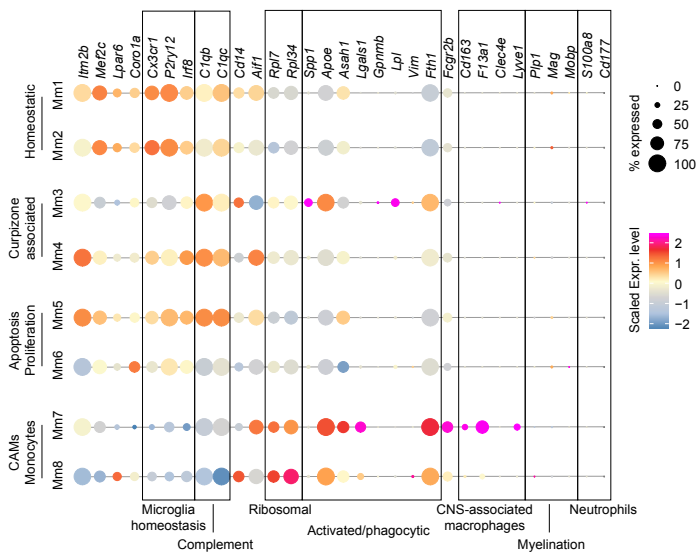**d**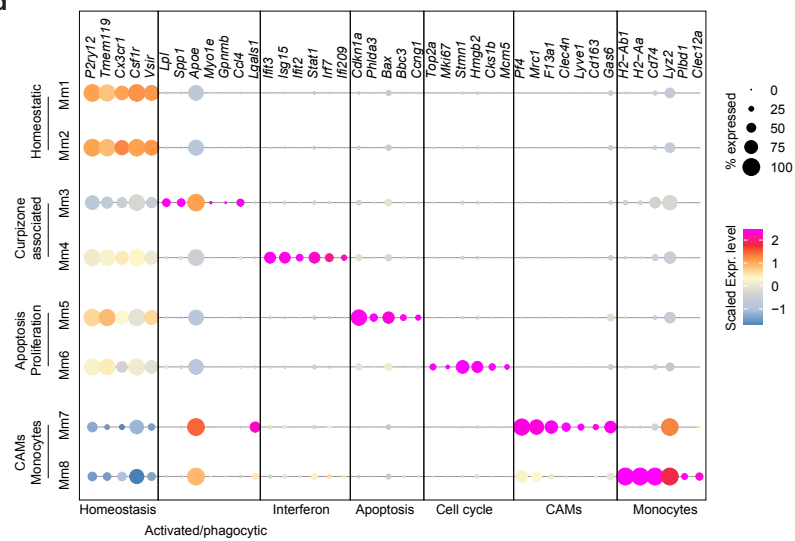

Supplement: Supplementary file 3 — Additional file 3: Figure S3. Supplementary data related to figure 3. (a) Volcano plot depicting significantly differentially expressed genes between Hs8 and Hs6 samples. (b) Bar plots depicting top 20 gene ontology terms associated with DEGs between Hs8 and Hs6 cells. (c) Heatmap depicting expression weighted gene set enrichment scores of microglia subcluster markers derived from Gerrits et al. (2021) in the human MS dataset. Asterisks depict significant enrichments (p < 0.05). Abbreviations: NACT = normal appearing cortical tissue; NAWM = normal appearing white matter; WML = white matter lesion. [file 40478_2021_1306_MOESM3_ESM.pdf]

Supplementary figure S3

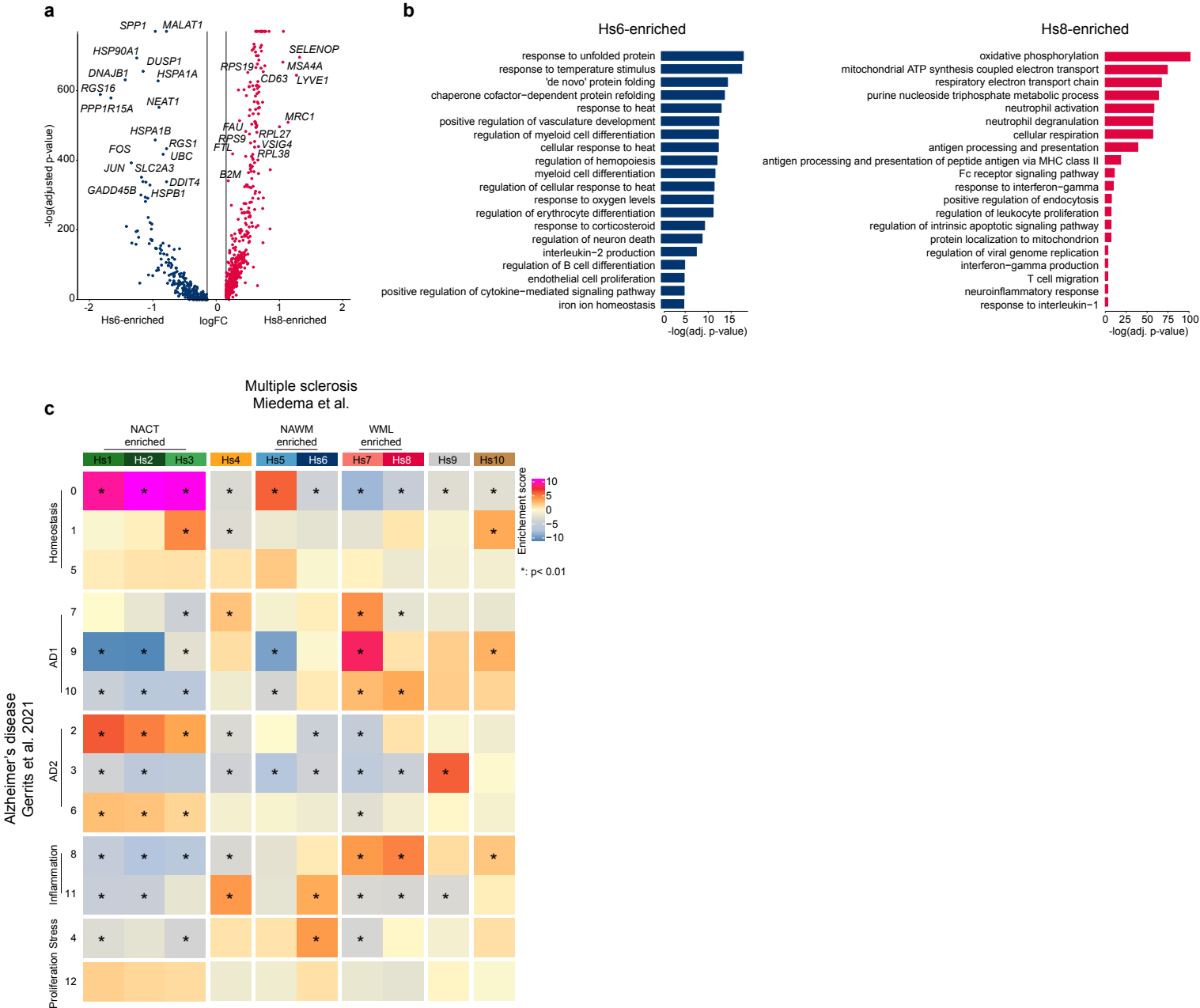

Supplement: Supplementary file 4 — Additional file 4: Figure S4. Supplementary data related to figure 4. (a) Representative plots depicting FACS strategy to obtain live Cx3cr1pos cells. (b) Boxplots per sample depicting several QC indicated stats per cell. (c) Dot plot depicting expression of mouse homologs of the genes depicted in Figure 3f. (d) Dot plot depicting expression of marker genes of each mouse scRNAseq cluster. Size of the symbols depicts the fraction of cells expression the gene, color scale depicts average expression level. Abbreviations: CAM = CNS-associated macrophages. [file 40478_2021_1306_MOESM4_ESM.pdf]
