# Supplementary material for: Brain macrophages acquire distinct transcriptomes in multiple sclerosis lesions and normal appearing white matter
Source: Acta Neuropathol Commun. 2022 Jan 28;10:8. doi: 10.1186/s40478-021-01306-3 (PMC8796391; doi:10.1186/s40478-021-01306-3)

Supplementary figure S5

a

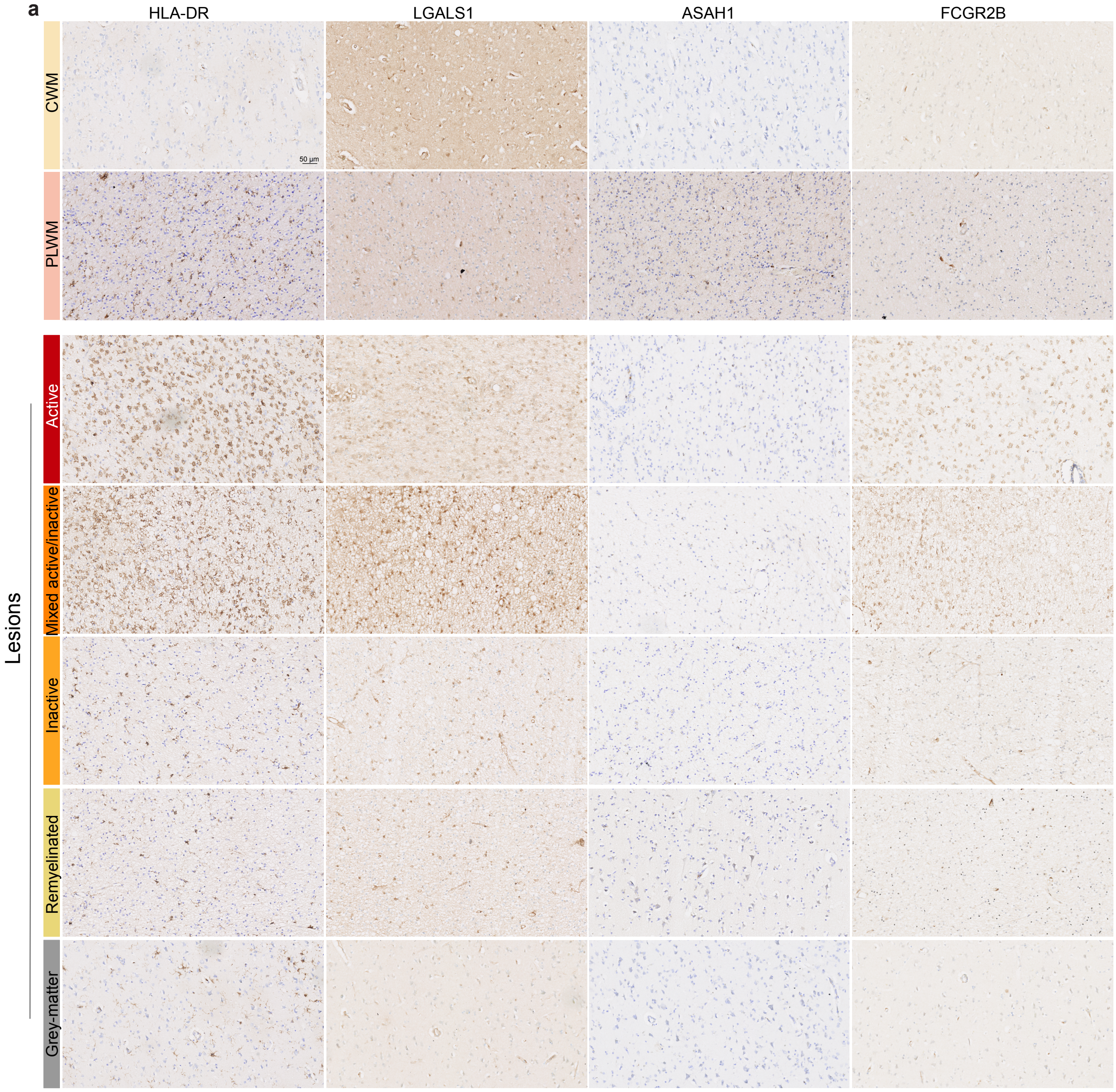

b

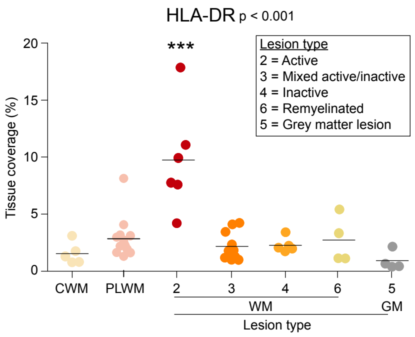

Supplement: Supplementary file 5 — Additional file 5: Figure S5. Supplementary data related to figure 5. Representative images of immunohistochemistry for HLA-DR, LGALS1, ASAH1 and FCGR2B in CTR donors and 6 lesion types in MS donors. Abbreviations: CWM = control white matter (CTR donor), PLWM = perilesional white matter. [file 40478_2021_1306_MOESM5_ESM.pdf]
